# Honokiol-mediated reversal of cisplatin-induced transcriptomic changes in outer hair cells

**DOI:** 10.1016/j.biopha.2026.119857

**Published:** 2026-08-19

**Authors:** Chail Koo, Robert Fuentes, Prerita Pandya, Satoe Takahashi, Devin Thomas, Yingjie Zhou, Kazuaki Homma, Jin Li, Claus-Peter Richter, Xiaodong Tan

**Affiliations:** a Department of Otolaryngology Head and Neck Surgery, Feinberg School of Medicine, Northwestern University, Chicago, IL, USA; b Department of Communication Sciences and Disorders, Northwestern University, Evanston, IL, USA; c Hugh Knowles Center for Clinical and Basic Science in Hearing and Its Disorders, Northwestern University, Evanston, IL, USA; d Department of Otolaryngology Head and Neck Surgery, Beijing TongRen Hospital, Capital Medical University, Beijing, China; e Department of Biomedical Engineering, Northwestern University, Evanston, IL, USA

**Keywords:** Cisplatin, Hearing Loss, Outer hair cells, Transcriptome, Oxidative stress, Mitochondria

## Abstract

Cisplatin-induced hearing loss (CIHL) affects half a million cancer patients annually, but treatment strategies are limited. We have previously shown that honokiol, an antioxidant and otoprotective compound, upregulates mitochondrial sirtuin 3 (SIRT3) and protects against CIHL. In the present study, we investigated the molecular requirements of this protection using a *Sirt3* knockout (*Sirt3*^−/−^) mouse model. Our results showed that honokiol pre-treatment ameliorated auditory threshold shifts in both wild-type and *Sirt3*^−/−^ mice. Quantitative histological analysis showed that honokiol pre-treatment protected against cisplatin-induced synaptopathy. To explore the underlying molecular mechanisms, we performed RNA–seq on wild-type outer hair cells (OHCs). Cisplatin treatment resulted in a global transcriptomic downregulation, which was biased towards longer genes. Differential expression analysis showed that cisplatin treatment resulted in the downregulation of mitochondrial redox genes, such as *Idh2* and *Aldh2*, a pattern that was reversed by honokiol pre-treatment. Computational transcription factor binding site analysis predicted that honokiol rescues chromatin-modifying pathways, while suppressing the ternary complex factor subfamily. Lastly, honokiol-only treatment proactively reinforced antioxidant defenses via *Sod2* upregulation. Together, these results provide a comprehensive transcriptomic map of OHCs in the cisplatin background and highlight the clinical potential of honokiol to protect against CIHL.

## 1. Introduction

Cisplatin-induced hearing loss (CIHL) is a major side effect of chemotherapy [1], affecting half a million cancer survivors worldwide each year [2]. Treatment for CIHL is limited due to concerns such as interference with cisplatin’s antitumor effect [3]. CIHL is associated with mitochondrial dysfunction induced by accumulation of reactive oxygen species (ROS) [4], such as hydrogen peroxide (H_2_O_2_) [5,6]. Elevated ROS levels indicate increased oxidative stress [7], leading to widespread cellular and structural damage within the cochlea [8,9]. Outer hair cells (OHCs) are particularly vulnerable to ROS [10–12].

We recently identified honokiol, a multifunctional polyphenol, as a promising candidate for treating CIHL without compromising cisplatin’s anti-tumor efficacy in a tumor-bearing mouse model [13]. Honokiol works through the activation of sirtuin 3 (SIRT3), a mitochondrial NAD^+^–dependent deacetylase [14]. SIRT3 is critical for mitochondrial function [15]; it activates antioxidant enzymes such as superoxide dismutase 2 (SOD2) [16] and isocitrate dehydrogenase 2 (IDH2) [17], which are both essential for neutralizing ROS [18]. In the cochlea, activation of SIRT3 protects against noise-induced [19,20], drug-induced [13,21], and age-related [22,23] hearing loss. In addition, SIRT3 overexpression protects inner hair cell (IHC) synapses against H_2_O_2_-induced oxidative stress in vitro, while H_2_O_2_ downregulates forkhead box protein O 3a (FOXO3a), SOD2, and IDH2 [24]. Recently, we further demonstrated that honokiol ameliorated an early-onset hearing loss in male mice lacking *Sirt3* [25]. This shows that additional molecular pathways [26–28], other than the SIRT3 pathway, are involved in honokiol’s otoprotective mechanism.

In the current study, we used a global *Sirt3* knockout (*Sirt3*^−/−^) mouse model to investigate the requirement of *Sirt3* for honokiol-mediated protection against CIHL. We investigated the otoprotection by measuring auditory thresholds, quantifying IHC synaptic integrity, quantifying SOD2 enzymatic activity, and quantifying oxidative stress by measuring cochlear H_2_O_2_ concentration. Furthermore, transcriptomic analysis of OHCs after honokiol and cisplatin treatments was also performed on wild-type controls to gain insights into the genes and pathways involved in CIHL and its protection.

## 2. Methods

### 2.1. Mouse strains and breeding

The Jackson Laboratory produced the mice by fertilizing oocytes (FVB/NJ females, #001800, RRID:IMSR_JAX:001800) with sperm (129-Sirt3tm1.1Fwa/J, #012755, RRID:IMSR_JAX:012755), generating a mixed 129/FVB background. The initial *Sirt3*^−/−^ breeding pairs and wild-type (*Sirt3*^+/+^) breeding pairs were from the progeny of *Sirt3* heterozygotes (*Sirt3*^+/−^). *Sirt3*^−/−^ mice have been used to study mitochondrial function in the past [25,29,30]. All mice appeared healthy with no developmental abnormalities. Genotyping was performed by TransnetYX (Cordova, TN) using ear tissue samples. Genomic DNA was obtained from ear tissues, and automated RT-qPCR was performed for the JAX #012755 strain using the two probes (Cre-excised mutant allele and wild-type allele) to determine homozygous mutant, heterozygous, and wild-type. Their genotypes were tested again using RT–qPCR at NUSeq Core Facility using custom primers, targeting between *Sirt3* exon 2 and exon 3. All breeders were homozygous for the mutant Cadherin 23 allele (Cdh23c.753 A) which is associated with progressive hearing loss beginning at 3–6 months of age [31,32]. To minimize confounding effects due to the mutation, auditory testing and euthanasia were performed before 90 days of age (Day 90).

### 2.2. Experimental group assignments and treatments

Mice were divided by genotypes (*Sirt3*^+/+^ or *Sirt3*^−/−^) and randomly assigned to drug treatment groups (vehicles, honokiol, cisplatin, or honokiol + cisplatin) at the age of 42 days. A three-cycle low-dose cisplatin treatment simulating a clinical chemotherapy paradigm was adopted [13,33,34]. In each cycle, four doses of honokiol (or corn oil) and cisplatin (or saline) were administered across four days (Days 43–46, 57–60, and 71–74), followed by a 10-day recovery interval [13, 33,34]. For the combination (honokiol + cisplatin) treatment group, honokiol was given one hour prior to cisplatin. Mice were euthanized after the experimental endpoint. Honokiol (J63434, Alfa Aesar, Ward Hill, MA) was dissolved in corn oil and administered via intraperitoneal (i.p.) injection at a dose of 10 mg/kg into the lower left abdomen. Cisplatin (P4394, Millipore Sigma, St. Louis, MO) was dissolved in sterile saline and administered via i.p. injection at a dose of 4 mg/kg.

For RNA–seq (for metadata, refer to GSE328003), only wild-type mice were used, receiving the same treatment doses: vehicles (*n* = 9), honokiol (*n* = 8), cisplatin (*n* = 8), or honokiol + cisplatin (*n* = 7). Treatments were given daily at postnatal day (P) 21–24. Mice were euthanized at P25.

No subjects or datapoints were excluded from the auditory tests, RNA–seq, and biochemical experiments. Animals which died or were euthanized during drug treatments were accounted for within the Kaplan-Meier survival model. In addition, investigators were blinded to the mouse genotypes and treatment groups during the auditory tests and quantitative analysis.

### 2.3. Auditory brainstem response measurement

Hearing was assessed by measuring ABR thresholds at Days 42, 56, and 84 (repeated measurements of the same mice) using Tucker-Davis Technologies (TDT) system (Alachua, FL). Mice were anesthetized by i.p. injection of ketamine (100 mg/kg; 1169507031, Covetrus) and xylazine (10 mg/kg; 5939911020, Akorn). Anesthesia was monitored every 15 min, and extra ketamine was given when it was required. Body temperature was maintained at 37 °C using a heating pad.

Tone bursts (plateau time 5 ms, rise/fall time 1 ms) were given through a MF1 speaker (TDT) pre-calibrated by Brüel & Kjær Nexus–2690-A–0S1 amplifier linked with a pressure field 1/8-inch microphone (Hottinger Brüel & Kjær A/S, Virum, Denmark). The frequencies delivered were 36, 32, 24, 16, and 8 kHz, and the sound level was between 85–20 dB sound pressure level (SPL) with a step of 5 dB. Responses were amplified by Medusa 4Z amplifier and recorded using RZ6 processor (TDT) using BioSigRZ software (Tucker-Davis Technologies, Alachua, FL, USA; RRID:SCR_014820). Attenuation was set at 21 dB and gain was at 0 dB. The response was defined as having an amplitude at least 1.5 times above the noise floor. Thresholds were defined as the lowest sound level eliciting a reproducible waveform in the averaged trace. Waveform data were converted into thresholds using custom MATLAB (MathWorks, Natick, MA, USA; RRID:SCR_001622) code, and visual inspection of the waveforms was followed to verify the generated thresholds. If there was no signal at the highest stimulation (85 dB SPL), the threshold was recorded as NaN.

### 2.4. Distortion product otoacoustic emission measurement

DPOAE thresholds were measured at Days 42, 70, and 84 using the TDT system. To optimize animal welfare and reduce mortality, we included a staggered design of ABR (Days 42, 56, 84) and DPOAE (Days 42, 70, 84) measurements. Anesthesia was performed in the same way as ABR measurements. F_1_ and F_2_ were given at a fixed ratio of F_2_/F_1_ = 1.22. The frequencies of F_2_ were set at 8, 16, 24, and 32 kHz. Stimulation levels were increased from 20 to 80 dB SPL with 10 dB increases to generate input/output functions. The cubic distortion product (2 F_1_–F_2_) was recorded and applied to custom MATLAB (RRID:SCR_001622) code. The amplitude at the 2 F_1_–F_2_ frequency was determined using the fast Fourier transform. The noise floor was determined by averaging the signal levels of frequency bins (*n* = 10) around the 2 F_1_–F_2_ peak. DPOAE thresholds were defined as the sound level of F_1_ (L_1_) at which DPOAE amplitude exceeded 0 dB SPL. If there was no signal at the highest stimulation (80 dB SPL), the threshold was recorded as NaN.

### 2.5. Survival analysis

Mouse survival was checked daily starting from the treatment initiation until the experimental end point. Animals with a weight loss greater than 25%, in critical conditions, or other physical ailments detrimental to long-term well-being were removed from the study, euthanized, and recorded as non-survivors. Survival was estimated using the Kaplan-Meier method and visualized using the survminer [35] package. Statistical differences among the treatment groups were determined using the log-rank test. Cox proportional hazards regression was used to calculate hazard ratios and to study interactions among genotypes and drug treatments.

### 2.6. Animal euthanasia and cochlea harvest

Mice were euthanized using i.p. injection of 0.15 mL Euthasol^®^ (pentobarbital sodium 390 mg/mL and phenytoin sodium 500 mg/mL; Virbac). The lack of response was confirmed by a toe-pinch test. Cardiac perfusion with 4% paraformaldehyde (15714S, AOL) was conducted through a scalp vein set (26704, Exelint), and it was followed by decapitation and cochlear extraction.

### 2.7. Immunostaining

Cochleae were extracted after the final auditory tests and were post-fixed in 4% PFA for 2 h at room temperature on a shaker. The cochleae were then decalcified in 10% ethylenediaminetetraacetic acid (EDTA) at 4 °C for 72 h on a rotator. After decalcification, cochleae were washed in phosphate buffered saline (PBS) and dissected into five segments (S1–S5) from the apex to base [25]. The S3 region [25], corresponding to a frequency range of 17.3–40.0 kHz, was used for immunostaining, as this region allowed comparative quantitative analysis across treatment groups [25].

Dissected cochleae were washed in 1 × PBS (200 uL, 10 min each) at room temperature. The samples were incubated in 30% sucrose solution in PBS for 15 min, frozen at −80 °C for 10 min, and thawed at room temperature for 15 min to increase tissue permeability. The samples were washed again in PBS. The samples were incubated for 1 h at room temperature in a blocking solution containing 20% goat serum (7332500, Lampire), 1% Triton X–100 (93443, Sigma-Aldrich; RRID: SCR_008988), and 1 × PBS.

The blocking solution was replaced with antibody solution (Table 1) prepared in fresh blocking buffer. The samples were incubated at 37 °C for 16 h. The tissues were washed in PBS (200 uL, 10 min each, 3 times). The tissues were incubated with secondary antibody solution (Table 1) at 37 °C for 2 h in the dark. The tissues were mounted on slides (48311703, VWR) using ProLong Gold Antifade Reagent (P36930, Invitrogen).

### 2.8. Confocal microscopy and synapse visualization

Confocal imaging took place at the Nikon Imaging Center, Northwestern University, using a Nikon A1 Confocal Laser Microscope equipped with a 100 × oil-immersion objective (NA 1.45). Images were captured at 512 × 512 pixels. Z-stacks had a 0.2 μm step size, starting from the top of CtBP2-positive nucleus region to the base of IHCs, where ribbons typically fade. The depth of imaging ranged from 25 to 35 μm, depending on the sample.

Consistent pinhole sizes (0.8 for 640 nm), pixel dwell time (4.8), HV, and offset (0) were used. Lasers with wavelengths (405, 488, 561, and 640 nm) were used for Hoechst, GluR2, CtBP2, and MYO7A signals, respectively. Images were acquired using NIS-Elements C software (Nikon) and processed in Fiji (ImageJ; RRID:SCR_002285). Under the Brightness/Contrast menu, Min and Max values were adjusted identically for each channel, after optimizing for the vehicle control images.

### 2.9. Cell and synapse counting

Confocal images were loaded in Fiji to horizontally orient the IHC row, and they were loaded onto Imaris (v9.2.1, Bitplane, Zurich, Switzerland; RRID:SCR_007370) for 3D analysis. In the Surpass view, a cuboidal region of interest (ROI) was selected to encompass IHC bodies, presynaptic ribbons, and postsynaptic densities, excluding OHC rows and IHC nuclei. CtBP2 and GluR2 spots were captured using a 0.5 μm diameter and background subtraction, with quality thresholds between 300–2000. Colocalized synapses were counted using Spots function, while orphan ribbons and densities were calculated by total spots – colocalized spots.

IHC and OHC survival was measured within the S3 segment (17.3–40.0 kHz) [25] of the cochlea. MYO7A signal was used to identify hair cell bodies. To count cells, 3D surface reconstruction was carried out using Imaris (v.9.2.1). In the Surpass view, ROI surrounding the OHC rows was defined, and OHCs were defined as MYO7A-positive cell bodies using the Spots function with an estimated diameter of 2.0 μm while background subtraction was enabled. Non-surviving OHCs were determined by the absence of MYO7A signal at expected positions, using the regular spacing of the three OHC rows as a reference. Cell survival percentage was calculated as (observed number of cells / expected number of cells) × 100. IHC survival was manually quantified using NIS-Elements C software. Significance was determined via one-way ANOVA followed by Tukey’s post-hoc test for multiple comparisons.

All quantifications were carried out without the knowledge of experimental groups. Data were presented as boxplots showing median, quartiles, and range.

### 2.10. Outer hair cell collection

Cochleae were collected from euthanized P25 mice and the bulla was removed. Each cochlea was placed in 1 × PBS solution (AM9624, Invitrogen). Cochlear bones were slowly removed starting from the tip of the apex, exposing the organ of Corti. The basal end of the coil was gently grasped, and the coil was removed from the base to apex in a circular motion. The removed coil was scooped with a micro curette and poured onto a petri dish containing a droplet of 50 uL Dispase (07913, STEM-CELL Technologies) at 5 U/mL. The coil was then incubated for 8 min at room temperature. The solution was diluted with 250 uL of cold PBS to stop the reaction. The suspension was triturated using a 200 μL low-binding pipette tip (TS-200L, Phenix Research Products). The petri dish was kept on ice for 5 min for the cells to settle. The petri dish was brought under a LEICA DMIRB microscope attached to a micromanipulator. OHCs were scanned using Micro Manager software (v1.4.22; RRID:SCR_000415) and collected using fine-tipped glass capillary pipettes (TW150–4, World Precision Instruments) (Fig. 1A). A total of 20 OHCs were collected into a 0.2 mL tube with the final volume of 5 μL (Fig. 1B). Tubes were immediately stored on dry ice.

A total of 32 mice across treatment groups were used for OHC collection, with one cochlea harvested per animal: Vehicle control (6 males, 3 females), Cisplatin (3 males, 5 females), Honokiol (5 males, 3 females), and Honokiol + Cisplatin (4 males, 3 females).

### 2.11. Processing extracted cells

Downstream processing of extracted OHCs was carried out at NUSeq Core Facility at Northwestern University (Fig. 1C). RNA extraction was not carried out due to the ultra-low input materials. Instead, complementary DNA (cDNA) synthesis was directly performed using Takara SMART-Seq mRNA (634773, Takara Bio), while the kit allowed immediate cell membrane lysis and release of cellular mRNA into solution. Library prep was performed using Illumina Nextera XT DNA Library Kit (15032354, Illumina). Library concentration was determined using Qubit 2.0 Fluorometer and Qubit dsDNA HS Assay Kit (Q32854, Thermo Fisher Scientific). Fragment distribution was determined using an Agilent 2100 Bioanalyzer with the High Sensitivity DNA Kit (50674626, Agilent). Sequencing was performed using Illumina NovaSeq X Plus and 50 bp paired-end reads with estimated 1.5 billion reads (Fig. 1C).

### 2.12. Transcriptome quantification

Raw paired-end sequencing reads were processed through a specialized transcriptomic pipeline (Fig. 1C). Transcript abundance was quantified using Salmon (v1.10.2) [36]. The transcriptome index was constructed from the Mus musculus GRCm39 assembly using a k-mer length of 31 and automated poly-A tail clipping. Selective alignment was used via validateMappings [37] to ensure high-fidelity quantification, by accounting for potential sequence biases and adapter contamination without the need for manual trimming. To test for OHC purity, we validated the enrichment of the OHC-specific marker oncomodulin (*Ocm*) across all 32 experimental samples; we compared the log_2_ normalized *Ocm* expression vs. the mean expression of non-OHC markers (*Sox2*, *Tubb3*, and *Kcnj10*) using a paired two-tailed *t*-test.

### 2.13. Differential expression

Transcript counts were imported using the tximport [38] package. Genes with low expression (< 10 reads in at least 3 samples) were excluded. Differential gene expression (DGE) analysis utilized DESeq2 (RRID:SCR_000154) [39] using (~ batch + treatment group) to account for variance (Tables S2–4). Metadata outlining sex, treatment groups, and batches are available in the GEO database (GSE328003). Batches indicate different library prep dates while all samples across the batches were sequenced together on the same run. For visualization, count matrices went through a variance-stabilizing transformation (VST). To extract biological effects from technical artifacts arising from library preparation, batch effects were minimized across the VST-transformed matrix using limma (RRID:SCR_010943) removeBatchEffect. PCA was done via prcomp function using the top 1000 DEGs (Figure S1). Initial exploratory analysis showed that sex did not significantly affect the global transcriptional variance. Specifically, variance partitioning via ANOVA on principal components showed the impact of biological sex on PC1 (0.99%, p = 0.31) and PC2 (0.39%, p = 0.61). Therefore, sex was excluded from the final design.

### 2.14. Pathway analysis

Gene set enrichment analysis (RRID:SCR_003199) [40] was carried out on genes which were ordered by the DESeq2 [39] Wald statistics using clusterProfiler (RRID:SCR_016884) [41] and the org.Mm.eg.db (RRID:SCR_025175) annotation. Gene ontology biological process terms were used. Final selection of the processes focused on mitochondrial pathways.

### 2.15. Gene expression plots

To visualize the otoprotective effect of honokiol, a reversal analysis was carried out. Reversed genes were defined as being differentially expressed by cisplatin (cisplatin vs. control), but modulated in the opposite direction by honokiol (honokiol + cisplatin vs. cisplatin) (*p* < 0.05 for both contrasts). The global effect of this reversal was estimated using Pearson correlation *r* of the log_2_FC across all protein-coding genes. Volcano plots were generated using ggplot2 (RRID: SCR_014601) [42] and ggrepel [43] in R (v4.4.2; RRID:SCR_001905).

### 2.16. Heatmap generation and gene filtering

To visualize the expression and clustering across biological replicates, supervised hierarchical clustering was performed using the ComplexHeatmap (v2.22.0) package. For downstream visualization, low-abundance transcripts were removed with a requirement of log_2_ normalized reads > 1 in at least 25% of the samples within the cohorts. Predicted genes, RIKEN transcripts, pseudogenes, and ribosomal genes (Gm-, -ps, Rps-, and Rpl-) were removed so that we could focus on protein-coding transcripts. Rows were divided into four clusters using the *k*-means (*k* = 4) to characterize the transcript blocks. Separate tracts were added to the right side of the plot using custom annotations indicating two things: a horizontal bar plot showing the mean base expression abundance (log_2_) for each gene and a list of exemplary gene names. For visualization using pheatmap [44], supervised selection was first used to extract reversal genes which were statistically significant differential expression (p < 0.05, DESeq2 Wald test) in both cisplatin vs. control and honokiol + cisplatin vs. control. Selected genes were first manually organized into predefined biological pathways without hierarchical row clustering based on mitochondrial function, redox biology, apoptosis signaling, autophagy, ion transport, and lipid metabolism. Log_2_FC values from both contrasts (cisplatin vs. control, and honokiol + cisplatin vs. cisplatin) were merged by the gene symbols.

### 2.17. Transcription factor binding site motif enrichment

To identify potential upstream regulators which drove the gene expression changes, we computationally carried out an unbiased TFBS motif enrichment analysis using the RcisTarget package (v1.22.0) in R. The DEGs were divided into two tracks based on the directional change of gene expressions across the drug treatment groups. Rescued track analyzed genes which were significantly downregulated by cisplatin but upregulated by honokiol pre-treatment, while the suppressed track analyzed genes which were significantly upregulated by cisplatin but downregulated by honokiol pre-treatment. To address the version difference between the RNA–seq (GRCm39/mm39) and the RcisTarget (mm9-tss-centered–10kb–7species.mc9nr.genes_vs_motifs.rankings. feather), input DEGs were put in directly using standard MGI gene symbols (gene_name) rather than genomic coordinates. The promoters were scanned for overrepresented cis-regulatory motifs around the transcription start site (TSS) [45]. The top 10 enriched transcription factors by normalized enrichment score (NES) were visualized using ggplot2 in R. An exemplary genomic locus trace was captured using the UCSC Genome Browser, showing the predicted transcription factor binding sites.

### 2.18. cDNA conversion and RT–qPCR

cDNA synthesis utilized SuperScript IV VILO (Fisher Scientific, 117660050). RT–qPCR was carried out in the cochleae of Day 90 mice across the treatment groups using PowerTrack SYBR Green Master Mix (Fisher Scientific, A46012). PCR reactions included no-RNA, no-RT, and no-cDNA controls with three technical replicates for each sample. Custom DNA oligos (Sigma, 41105327) were used and the sequences are available in Table S1. Gene expression for RT–qPCR utilized the ΔΔCt method [25], in which 2^^−(ΔΔCt)^^ was used to describe the expression change.

### 2.19. Protein quantification

Total protein concentration was calculated using the Micro BCA Protein Assay Kit (Thermo Fisher Scientific, 23235). For Day 90 mice, cochleae were homogenized in HEPES buffer (HEPES, EGTA, D-mannitol, and sucrose) and were kept at 4 °C in the dark. Two technical replicates were run on flat-bottom microplates (Fisher Scientific, 351172). A standard curve was established using bovine serum albumin. After 2 h at 37 °C, absorbance was measured at 562 nm using a Synergy HTX Multi-mode reader with Gen5 3.11 software. Protein concentrations were calculated using (OD_562_–y-intercept) / slope.

### 2.20. SOD2 activity assay

Mitochondrial SOD2 activity was measured using the Cayman Superoxide Dismutase Assay Kit (Cayman Chemical, 706002). Homogenates from one cochlea were centrifuged at 1500 × g. The supernatant was normalized for protein concentration. To obtain mitochondrial SOD, samples were centrifuged at 10,000 × g, and the supernatant was removed and pellets were resuspended in HEPES buffer. Superoxide radicals were generated using xanthine oxidase and the reaction was started. After 30 min at room temperature, absorbance was measured at 450 nm. SOD2 activity (U/mL) was measured by using a standard curve and the kit-specified formula = [(linearized rate – y-intercept) / slope] × (0.23 / 0.01).

### 2.21. H_2_O_2_ quantification

Using cochleae extracted from Day 90 mice, cochlear H_2_O_2_ concentrations were measured using Peroxide Assay Kit (Sigma-Aldrich, MAK311). It uses the oxidation of Fe^2+^ to Fe^3+^ and the reaction with xylenol orange. Homogenates from one cochlea were centrifuged at 10,000 × g, and the supernatant was immediately used for downstream processes in the dark. Samples and H_2_O_2_ standards were incubated with a detection reagent for 30 min at room temperature. Absorbance was measured at 585 nm. Concentration (μM) was calculated as: (OD_585_–y-intercept) / slope.

### 2.22. Sex as a biological variable

For auditory tests and biochemical experiments, we exclusively investigated male mice because our prior work [25] showed that males are uniquely vulnerable to moderate hearing loss due to *Sirt3* deficiency, while females do not reach a statistically significant difference.

### 2.23. Statistics

ABR thresholds were analyzed using linear mixed-effects models (lme4 [46] and lmerTest [47]), with genotype, days (weeks), frequency, and treatment group included as fixed effects and mouse ID as a random effect. Pairwise comparisons among treatment groups were performed using estimated marginal means (emmeans [48]). *P*-values were adjusted using the Benjamini–Hochberg false discovery rate (FDR) procedure. Data were reported as mean ± standard deviation. For survival analysis, pairwise comparisons among treatment groups used pairwise_survdiff [35] function with *p*-values adjusted using FDR. All analyses were carried out in R (v4.4.2; RRID:SCR_001905). For RT–qPCR, Two-way ANOVA (for *Idh2*) or one-way ANOVA (for *Sirt3*) were followed by Tukey’s post-hoc test for multiple comparisons. For SOD2 assay and H_2_O_2_ quantification, statistical significance was determined by two-way ANOVA followed by Tukey’s post-hoc test.

Group sizes for ABRs and DPOAEs were determined based on established variance and effect sizes shown in our previous studies [13,25] to ensure statistical power (1 −β > 0.80) to detect a minimum of 10 dB in auditory thresholds. Sample sizes for RNA–seq were determined using a published guideline [49]. Our sample sizes (*N* = 32, *n* ≥ 7 biological replicates per group) passed the minimum threshold of *n* = 6 to achieve > 85% sensitivity for detecting mid-to-high effect DEGs.

## 3. Results

### 3.1. Honokiol attenuates cisplatin-induced ABR threshold elevation in both Sirt3^+/+^ and Sirt3^−/−^ mice

To evaluate cisplatin-induced damage and honokiol-mediated protection, baseline auditory brainstem responses (ABRs) were assessed at Day 42, and drug treatments began at Day 43, involving four male treatment groups: vehicles, honokiol, cisplatin, and honokiol pre-treatment + cisplatin. Treatments lasted for four consecutive days, followed by ten days of recovery (Fig. 2A). Across two different genotypes (*Sirt3*^+/+^ & *Sirt3*^−/−^), we compared two treatment groups, honokiol + cisplatin vs. cisplatin, to investigate honokiol-induced effect in the cisplatin background.

At baseline (Day 42) without any drug treatment, no significant differences were observed in ABR thresholds at any frequency in either genotype (*p* > 0.05) (Fig. 2B, Baseline).

At cycle 1 (Day 56), honokiol + cisplatin significantly ameliorated threshold shifts in both genotypes compared to cisplatin-only; in *Sirt3*^+/+^ mice, significantly decreased thresholds were observed at 32 kHz (mean difference = 6.61 dB, *p* = 0.019) and 36 kHz (mean difference = 13.05 dB, *p* = 8.76 × 10^−7^) (Fig. 2B, Cycle 1). Similarly, in *Sirt3*^−/−^ mice, honokiol pre-treatment significantly decreased thresholds at 8 kHz (16.44 dB, *p* = 1.18 × 10^−6^), 24 kHz (18.50 dB, *p* = 7.82 × 10^−8^), 32 kHz (9.47 dB, *p* = 0.004), and 36 kHz (12.41 dB, *p* = 2.24 × 10^−4^).

At cycle 3 (Day 84), CIHL was observed across most frequencies in both genotypes, and was alleviated by honokiol pre-treatment (Fig. 2B, Cycle 3). In *Sirt3*^+/+^ mice, significant decreases in threshold shifts were observed at all tested frequencies (8–36 kHz; all *p* ≤ 0.003). In *Sirt3*^−/−^ mice, compared to cisplatin-only, honokiol + cisplatin significantly decreased thresholds at 8 kHz (20.60 dB, *p* = 1.74 × 10^−9^), 16 kHz (10.80 dB, *p* = 0.001), 24 kHz (8.74 dB, *p* = 0.009), and 32 kHz (17.12 dB, *p* = 3.97 × 10^−7^). No significant difference was seen at 36 kHz (*p* = 0.68).

Overall, these findings showed that honokiol pre-treatment provided protection against CIHL in both *Sirt3*^+/+^ and *Sirt3*^−/−^ mice. Global analysis using a linear mixed-effects model identified significant interactions among genotype, frequency, and treatment for the ABR thresholds (*p* < 0.001).

### 3.2. Honokiol reduces cisplatin-induced DPOAE threshold elevations in both genotypes

OHCs are one of the primary targets of cisplatin-induced damage [11]. Therefore, we measured distortion product otoacoustic emissions (DPOAEs) to evaluate the functional integrity of OHCs. At baseline (Day 42), no significant differences were observed between honokiol + cisplatin vs. cisplatin at any frequency in either genotype (all *p* > 0.05) (Fig. 2C, Baseline). At cycle 2 (Day 70), honokiol pre-treatment significantly decreased the cisplatin-induced threshold shifts in both genotypes (Fig. 2C). In *Sirt3*^+/+^ mice, the decreases were observed at 8 kHz (mean difference = 5.76 dB, *p* = 0.010) and 16 kHz (5.55 dB, *p* = 0.010), with no significant change at 24 kHz (*p* = 0.112). Similarly, in *Sirt3*^−/−^ mice, honokiol pre-treatment significantly decreased threshold shifts at 16 kHz (8.45 dB, *p* = 0.0024) and 24 kHz (13.21 dB, *p* = 2.02 × 10^−6^), with no significant change at 8 kHz (*p* = 0.124). By cycle 3 (Day 84), the protective effect of honokiol pre-treatment was still present (Fig. 2C, Cycle 3). In *Sirt3*^+/+^ mice, significant decreases in threshold shifts were observed at 8 kHz (8.94 dB, *p* = 3.43 × 10^−5^) and 24 kHz (13.16 dB, *p* = 1.59 × 10^−9^), while threshold shifts at 16 and 32 kHz did not reach statistical significance. In *Sirt3*^−/−^ mice, honokiol + cisplatin, compared to cisplatin alone, significantly decreased thresholds at 16 kHz (8.37 dB, *p* = 0.0027) and 24 kHz (18.73 dB, *p* = 8.08 × 10^−12^). No significant differences were detected at 8 or 32 kHz.

Overall, these findings showed that honokiol protected the OHC function following cisplatin treatment in both *Sirt3*^+/+^ and *Sirt3*^−/−^ mice. While protective effects were shown in both genotypes, the frequency range and pattern of protection differed slightly.

### 3.3. Honokiol modifies mouse survival in the cisplatin background

We used Kaplan-Meier analysis to investigate the effect of cisplatin treatment on mouse survival and determine whether honokiol pretreatment offered mitigation. Cisplatin treatments on Days 42, 56, and 70 resulted in different overall survival; cisplatin-only group (wild-type) showed a survival rate of 75% by Day 90 (Fig. 3A) and cisplatin-only group (*Sirt3*^−/−^) showed a survival rate of 54.5% by Day 90 (Fig. 3B), while the pairwise log-rank test showed non-significance (*p* = 0.299). Global log-rank test showed significant difference of survival among the four treatment groups (*p* = 0.006), while the comparison between honokiol + cisplatin vs. cisplatin did not show statistical significance (*p* = 0.451). Cox proportional hazards regression showed no significant interaction among genotypes and drug treatments (*p* = 0.632). In summary, these outcomes show that honokiol pre-treatment does not significantly change the overall survival.

### 3.4. Honokiol protects against cisplatin-induced synaptopathy by preserving postsynaptic densities

CIHL is associated with IHC synaptopathy [33,50]. Using confocal microscopy, we verified in wild-type mice if cisplatin treatment results in reduction of IHC afferent synapses in the S3 segment (17.3–40.0 kHz) [25] and if honokiol pre-treatment mitigates it (Fig. 4; see Figure S2 **for unadjusted images**). We quantified the presynaptic ribbons (CtBP2-positive) (Fig. 5A) and postsynaptic densities (GluR2-positive) (Fig. 5B) per IHC. We focused on two comparisons: cisplatin vs. control (to investigate cisplatin-induced effect) and honokiol + cisplatin vs. cisplatin (to investigate honokiol-induced effect in the cisplatin background).

Quantitative analysis of the presynaptic ribbons showed that cisplatin treatment did not result in a significant change in CtBP2 puncta per IHC, compared to controls (*p* = 0.058) (Fig. 5A). There was a mean difference of −4.10, and high variability across samples was detected (SE of 2.02), while CtBP2 puncta in cisplatin group (Fig. 4G) looked visually dimmer than controls (Fig. 4A). This suggests that the cisplatin-induced change in presynaptic ribbons is highly heterogeneous. Pre-treatment with honokiol also did not affect the CtBP2 puncta count (*p* = 0.614) (Fig. 5A).

In contrast, the GluR2 puncta showed a significant loss of 57.6% in the cisplatin group compared to controls (6.67 vs. 15.74, *p* < 0.0001) (Fig. 5B). Pre-treatment with honokiol showed a 71.1% increase of GluR2 puncta compared to the cisplatin-only group (11.41 vs. 6.67, *p* = 0.0039) (Fig. 5B). The colocalized puncta count was also higher in the honokiol + cisplatin group compared to cisplatin-only group (9.73 vs. 5.56, *p* = 0.013) (Fig. 5C). Overall, these findings suggest that cisplatin treatment damaged the postsynaptic component of ribbon synapses, while honokiol pre-treatment protects this cisplatin-induced synaptopathy.

### 3.5. Honokiol prevents cisplatin-induced outer hair cell loss while inner hair cells remain unchanged

Across 22 mice, we observed an average of 189.5 ± 8.1 IHCs and 563.6 ± 57.5 OHCs in the S3 segment (17.3–40.0 kHz) (Table S1). We compared the survival of hair cells between cisplatin vs. control (to investigate cisplatin-induced effect), honokiol + cisplatin vs. cisplatin (to investigate honokiol-induced effect in cisplatin background). IHC counts remained unchanged across the treatment groups (Fig. 5D) and maintained normal morphological appearances (Fig. 4Q–T). Cisplatin treatment alone resulted in a decrease of OHC survival, compared to the control group (75.3% vs. 97.3% survival; *p* < 0.0001) (Fig. 5E). This quantitative change reflected the missing MYO7A signals within the OHC rows (Fig. 4S, red arrows). Honokiol pre-treatment partially reversed this damage, resulting in an increased OHC survival compared to cisplatin alone (88.8% vs. 75.3% survival; *p* = 0.0008) (Fig. 5E). Overall, cisplatin treatment resulted in moderate OHC loss, while honokiol pre-treatment ameliorated it.

### 3.6. RNA–seq analysis

To investigate the molecular mechanisms of CIHL and honokiol-mediated protection, we carried out RNA–seq on isolated OHCs. Analysis of FASTQ files (GSE328003, *n* = 32 samples) showed that Phred scores exceeded 34, indicating base-calling accuracy of 99.96%. The libraries showed a stable GC content distribution of 45–50% and negligible adapter contamination, showing that, despite the low starting number of cells (*n* = 20 cells per sample), the library complexity was preserved. Principal component analysis (PCA) showed that PC1 accounted for 41.4% of the total biological variance, capturing the drug treatment effects, while PC2 accounted for 5.6% of the variance (Figure S1).

OHC enrichment in collected samples was investigated by comparing log_2_-normalized expression of *Ocm* to non-hair cell markers (*Sox2*, *Tubb3*, and *Kcnj10*) within each sample. Across the 32 samples, *Ocm* expression was higher than the mean expression of the non-OHC markers by 11.63 ± 4.38 log_2_ units (95% CI: 10.05–13.20). This difference was significant (paired two-tailed *t*-test, *p* = 8.86 × 10^−16^). Cisplatin treatment (cisplatin vs vehicle) did not significantly change the *Ocm* expression in OHCs (log_2_ fold change (log_2_FC) = 1.59; *p* = 0.058).

### 3.7. Global exploratory analysis

We first investigated the wide transcriptional effect of drugs using a FC threshold of |log2FC= > 0.2. Each treatment (cisplatin or honokiol) resulted in genome-wide changes, showing a preference toward gene downregulation (Fig. 6A–B). In OHCs exposed to cisplatin vs. vehicle controls, among 18,196 transcripts with directional changes, 11,753 genes (64.59%) showed downregulations. Similarly, honokiol treatment resulted in global transcriptomic suppression; among the 18,822 genes with directional changes, 10,237 genes (54.39%) were downregulated, and 8585 genes (45.61%) were upregulated. Therefore, we established that the two drugs individually can trigger directional responses. Next, we investigated whether these gene expression changes are associated with gene lengths. Interestingly, we found a significant negative transcriptional bias depending on the gene length, for both cisplatin and honokiol (Fig. 6C). Cisplatin showed global transcriptional suppression as gene length increased (*R* = −0.021, *p* = 0.0064). A similar negative correlation was seen under honokiol treatment (*R* = −0.099, *p* = 2.2 × 10^−16^). These global changes showed that mouse genomic transcripts between 10 ~ 100 kb are sensitive to drug-induced transcriptional changes. We then investigated the combination therapy group, comparing honokiol + cisplatin vs. cisplatin, to capture the specific otoprotective mechanism of honokiol. Interestingly, the negative correlation was no longer present (*R* = 0.012, *p* = 0.12). Additionally, in the combination group, there were a number of high-magnitude expressions with log_2_ fold changes of + 30 and −30. This showed that honokiol under the cisplatin background drives high-magnitude functional changes for specific genes.

To visualize the drug-induced changes and identify patterns, we performed supervised hierarchical clustering using the top 1000 variable protein-coding genes across the three experimental groups (Fig. 7). We demonstrated that individual biological replicates show clustering with columns being segregated by the three treatment groups: control (vehicles), cisplatin, and honokiol + cisplatin. The resulting blocks show a pattern that genes which were turned off by cisplatin were turned back on by honokiol pre-treatment, while genes which were turned on by cisplatin were blocked. This systematic pattern suggests that honokiol can negate the transcriptional changes induced by cisplatin to an extent.

### 3.8. Selective reversal of mitochondrial and stress response genes by honokiol

Next, differential expression analysis (*p* < 0.05, DESeq2 Wald test) was carried out, comparing cisplatin vs. controls, and honokiol + cisplatin vs. cisplatin (Tables S2–3). We identified 3235 significant differentially expressed genes (DEGs) (*p* < 0.05) following cisplatin treatment (Fig. 8A–B). Honokiol pre-treatment reversed 59.8% of all cisplatin-induced changes with a negative correlation (r = −0.66, *p* < 0.001) (Fig. 8D, Figure S3). Cisplatin-induced transcriptomic changes (cisplatin vs. control; Table S2), transcriptomic changes by honokiol pre-treatment (honokiol + cisplatin vs. cisplatin; Table S3), and the baseline effects of honokiol (honokiol vs. control; Table S4) describe respective transcriptomic mapping.

According to the pathway analysis (Fig. 8C), mitochondrial genes showed significant changes in gene expressions due to honokiol pre-treatment in the cisplatin background. *Idh2* and *Aldh2* expressions, important for mitochondrial redox and reactive aldehyde detoxification respectively, were significantly decreased in cisplatin vs. controls (Fig. 8A). This pattern was reversed in honokiol + cisplatin vs. controls (Fig. 8B). Mitochondrial stress regulators *Dele1* and *Atad3a* showed similar reversal patterns (Fig. 8A–B). *Atg7* (autophagy-related) and *Ogg1* (DNA repair-related) also showed directional changes (Fig. 8A–B). Among the expressed protein-coding genes, the negative correlation between cisplatin-induced and honokiol pre-treatment–induced changes was significant (Pearson *r* = −0.66, *p* < 0.001) (Fig. 8D). This pattern pointed to honokiol-mediated recovery from cisplatin-induced damage, rather than random changes.

### 3.9. Targeted transcriptomic analysis reveals honokiol-mediated restoration of mitochondrial and survival pathways

Next, we categorized the ‘reversal’ genes identified in Fig. 8D to assess their functional implication. The reversed genes were defined as having been significantly altered by cisplatin (*p* < 0.05) and subsequently significantly altered in the opposite direction by honokiol pre-treatment (*p* < 0.05). This filtering showed that the reversed genes were involved in important biological pathways (Fig. 9). Reversal pattern was observed in mitochondrial redox homeostasis (Group I) and electron transport chain stability (Group II) (Fig. 9); honokiol + cisplatin ameliorated the cisplatin-induced suppression of *Idh2* and *Aldh2*, which are essential for neutralizing ROS (Fig. 9). In addition, honokiol + cisplatin reversed the upregulation of mediators involved in DNA damage and apoptotic signaling (Group IV) and stress response (Group V), including *Atad3a* and *Dele1* (Fig. 9). There was also a significant reversal of expression in genes associated with ion transport (Group VI) and lipid metabolism (Group VII) (Fig. 9). Their involvements suggest that the function of honokiol is broader than just antioxidant defense in OHCs.

### 3.10. TFBS enrichment shows rescued and suppressed networks

To investigate the upstream regulation driving changes in gene expression, we computationally carried out an unbiased transcription factor binding site (TFBS) motif enrichment analysis using RcisTarget on the DEGs. Conserved genomic binding motifs were investigated if they appeared in the 10 kb windows around the TSS of the DEGs.

Within the group of gene expressions rescued by honokiol pre-treatment, we observed overrepresentation of structural and epigenetic regulators (Fig. 10A). The histone regulator *Hdac2* (NES = 4.15) and the chromatin-looping protein *Ctcf* (NES = 4.09) were relatively highly enriched, and it was followed by the nuclear receptor *Rxra* (NES = 3.47). *Ctcf* is important for baseline survival, as it allows active transcription via chromatin structure modification [51]. Cisplatin may disrupt this chromatin structure, leading to silencing of important antioxidant genes such as *Aldh2*. Our computational prediction suggests that honokiol pre-treatment in the cisplatin background counteracts this silencing effect at the mRNA level (Fig. 8D). This rescue is highlighted in the UCSC genome browser track, showing the potential *Ctcf* binding sites across the *Aldh2* genomic region (Fig. 10C).

In contrast, the networks which were increased by cisplatin, but decreased by honokiol, occurred in the ternary complex factor subfamily, including stress response regulators *Elk1* (NES = 4.07), Elk3 (NES = 3.80), and *Elk4* (NES = 4.46) (Fig. 10B). Since the *Elk* family is involved in the c-Jun N-terminal kinase (JNK) and MAPK stress pathway [52], this hints that cisplatin may drive the transcriptional activation of pro-apoptotic genes, while honokiol drives the reversal, ensuring the cell survival.

### 3.11. SIRT3-Dependent Maintenance of Cochlear Idh2 Expression

We previously showed the honokiol-induced SIRT3 upregulation in the cochlea using immunostaining [13,25]. Here, we further investigated the mRNA expression of *Sirt3* (Fig. 11A) and its downstream target, *Idh2*, using reverse transcription quantitative polymerase chain reaction (RT–qPCR) (Fig. 11B). Cisplatin vs. control did not result in a significant change of *Sirt3* expression (*p* = 1.00) (Fig. 11A). Honokiol + cisplatin vs. cisplatin also did not result in a significant change (*p* = 1.00) (Fig. 11A). *Sirt3* mRNA expression was not detected in any *Sirt3*^−/−^ groups (Fig. 11A).

We next investigated whether the protective effect of honokiol was associated with cochlear expression of *Idh2*, a downstream antioxidant effector of honokiol and *Sirt3*. In wild-type mice, cisplatin vs. control showed a significant decrease of *Idh2* mRNA levels (*p* = 0.005) (Fig. 11B). Strikingly, honokiol + cisplatin vs. cisplatin showed a reversal of *Idh2* expression, restoring it to levels significantly higher than the cisplatin-only group (*p* < 0.0001) (Fig. 11B). Two-way ANOVA for *Idh2* expression showed a significant interaction between genotype and treatment (*p* = 0.016). To see if the transcriptional reversal in *Idh2* depended on SIRT3, we investigated *Idh2* levels in *Sirt3*^−/−^ cochleae. Cisplatin vs. control did not result in a significant difference of *Idh2* expression (*p* = 0.222) (Fig. 11B). Honokiol + cisplatin vs. cisplatin also did not result in a significant difference of *Idh2* expression (*p* = 0.998) (Fig. 11B). Overall, these results suggest that SIRT3 deficiency downregulated honokiol-mediated expression of *Idh2*.

### 3.12. Honokiol attenuates cisplatin-induced oxidative stress in the cochlea

We investigated how honokiol ameliorates cisplatin-induced damage by quantifying enzymatic response and oxidative stress in *Sirt3*^+/+^ and *Sirt3*^−/−^ cochleae. We first investigated the enzymatic activity of SOD2, an important antioxidant enzyme which is activated by SIRT3-mediated deacetylation. Cisplatin treatment did not result in a significant change in SOD2 activity between cisplatin vs. control as well as honokiol + cisplatin vs. cisplatin (Fig. 12A). This non-significance was observed in both wild-type and *Sirt3*^−/−^ cochleae. These results show that the honokiol’s protective effect against CIHL may not depend on SOD2 enzymatic activity.

We then investigated the concentration of H_2_O_2_ as a measure of cochlear ROS accumulation (Fig. 12B). Cisplatin treatment resulted in increased H_2_O_2_ levels compared to controls in both wild-type (*p* < 0.01) and *Sirt3*^−/−^ (*p* < 0.0001) mice. Increased H_2_O_2_ levels after cisplatin treatment were higher in *Sirt3*^−/−^ mice, showing that SIRT3 deficiency may exacerbate oxidative stress. Pre-treatment with honokiol ameliorated cisplatin-induced increases in H_2_O_2_ levels in both wild-type and *Sirt3*^−/−^ groups. Overall, these results show that honokiol pre-treatment may ameliorate cisplatin-induced oxidative stress in the cochleae, while the antioxidant enzymatic potential of SOD2 remains unchanged.

### 3.13. Honokiol alone induces mixed responses in outer hair cells

Next, we investigated the effect of honokiol without the cisplatin background (i.e. honokiol vs. control) using our RNA–seq data. To see the baseline differences across the two treatment groups, we mapped the top 1000 variable genes (Fig. 13). The samples clustered into two distinct groups, showing that honokiol alone changes the OHC transcriptome. Honokiol-induced expression of important antioxidant genes such as isocitrate dehydrogenase subunit gamma (*Idh3g*), *Sod2*, and microsomal glutathione S-transferase 1 (*Mgst1*) suggest that honokiol may strengthen the antioxidant capability in OHCs even without the presence of cisplatin. Next, we carried out DEA. Honokiol treatment resulted in a large change in the OHC transcriptome, with 6987 DEGs (*p* < 0.05). There was a significant upregulation of *Sod2* (log_2_FC = 1.90, *p* = 0.0086), an important antioxidant enzyme. This shows that honokiol may increase the antioxidant potential in OHCs. Interestingly, *Sod2* expression was not significantly changed in cisplatin vs. control (*p* = 0.262), or honokiol + cisplatin vs. cisplatin (*p* = 0.511) in our previous DEA (Fig. 8). Peroxisome proliferator-activated receptor coactivator 1-alpha (*Ppargc1a*), involved in mitochondrial biogenesis, was significantly downregulated (log_2_FC = −1.55, *p* = 0.031) by honokiol compared to control, with no significant changes in cisplatin vs. control or honokiol + cisplatin vs. cisplatin. Overall, these results suggest that honokiol alone induces different responses, compared to the effects of honokiol in the cisplatin background.

## 4. Discussion

In this study, we investigated the roles of SIRT3, a mitochondrial enzyme, in honokiol-mediated protection against CIHL. Our longitudinal ABR (Fig. 2B) and DPOAE (Fig. 2C) measurements showed that honokiol pre-treatment attenuated cisplatin-induced threshold shifts. Interestingly, honokiol pre-treatment resulted in hearing protection in both wild-type and *Sirt3*^−/−^ mice (Fig. 2B–C), although past research indicated that honokiol works via SIRT3 activation [14]. At the cellular level, honokiol-induced protection was linked to increased OHC survival; 75.3% survival in cisplatin treatment group and 88.8% survival in honokiol + cisplatin treatment group. At the sub-cellular level, cisplatin treatment resulted in a 57.6% decrease of GluR2 puncta count, while honokiol pre-treatment partially ameliorated this loss (Fig. 5B). Honokiol-mediated upregulations of *Sirt5* mRNA and SIRT5 protein were previously reported in *Sirt3*^−/−^ cochleae [25], while *Sirt3* was found to be non-essential in the recovery of noise-induced hearing loss [19]. These pieces of evidence suggest that, although honokiol-mediated activation of SIRT3 is helpful [20,21], honokiol may also benefit hearing in alternative biological pathways using different sirtuin family members.

To better understand the mechanisms of honokiol-mediated otoprotection, we investigated at the molecular level the potential genes and pathways involved in CIHL with and without honokiol treatment. Our exploratory analysis of global gene expression in OHCs showed a structural pattern in the genome; both cisplatin and honokiol treatments resulted in a significant negative gene expression bias as the gene length increased (*R* = −0.021 and *R* = −0.099, respectively), while downregulating global gene expression (Fig. 6C). This transcriptional suppression on longer genes is similar to what has been seen in past research studies which investigated DNA damaging agents such as ultraviolet light, benzopyrene, and cigarette smoke [53,54]. Mechanistically, longer genes are under greater threat of RNA polymerase II stalling and elongation arrest [53]. What was remarkable in our study was that this length-dependent negative correlation disappeared on the combination therapy group (Fig. 6C). This suggests that honokiol specifically targets transcriptional downregulation induced by cisplatin.

Next, we visualized honokiol’s effect in the cisplatin background using the supervised hierarchical clustering of the top 1000 variable protein-coding genes (Fig. 7). The partitions across biological replicates showed that the essential genes (1st block from top) were turned off by cisplatin and driven back up by honokiol pre-treatment. In addition, clusters containing pro-apoptotic pathways (4th block from top) were activated by cisplatin, but they were blocked by honokiol pre-treatment. The top 1000 variable gene expression in the honokiol + cisplatin group resembled the pattern seen in the control group (1st and 4th blocks from top). Overall, the heatmap hints that honokiol pre-treatment returns cisplatin-induced transcriptomic changes back to the control level.

While explorative transcriptomic analysis showed global changes across the dataset, we next performed a formal DEA to identify statistically significant changes. Interestingly, honokiol pre-treatment brought changes in the expression of genes affecting mitochondrial functions. One important reversal was the downregulation of *Idh2* (log_2_FC = −4.87, *p* < 0.01) in cisplatin vs. control, and the upregulation of *Idh2* (log_2_FC = +5.02, *p* < 0.001) in honokiol + cisplatin vs. cisplatin-only (Fig. 8A–B). IDH2 is a mitochondrial enzyme activated by SIRT3 [17], which is important for cochlear antioxidant defense [55, 56]. IDH2 catalyzes the reduction of NADP^+^ to NADPH, protecting cells from ROS, which can induce apoptosis [56,57]. This pathway is highlighted by previous research showing that the loss of *Idh2* accelerates age-related hearing loss in male mice, in which there is increased apoptosis and DNA damage in the hair cells and the spiral ganglion neurons [57]. However, our analysis additionally highlighted pathways such as ion transport and lipid metabolism (Fig. 9). This showed that honokiol’s otoprotective mechanism may extend beyond the traditional antioxidant scavenging role via SIRT3 activation.

RNA–seq studies in CIHL have been performed in the past, varying in cell types and cochlear locations [58,59]. One RNA–seq study using HEI-OC1 cells after cisplatin treatment identified upregulations of apoptotic and cell-cycle arrest genes such as *Cdkn1a*, *Trp53inp1*, and *Fas*, and inflammatory genes such as *Txnip* [58]. Interestingly, our OHC dataset lacked these signals, but showed downregulations of antioxidant genes such as *Sod3*, *Aldh2*, *Idh1*, *Idh2*, *Gpx5*, and *Gpx7* (Table S2). These discrepancies highlighted the necessity to verify transcriptomic data *in vivo*, while our studies might point more towards early cisplatin-induced changes before major apoptotic events. Previously, a single cell RNA–seq (scRNA–seq) study was performed on stria vascularis following acute high-dose cisplatin exposure [59]. Genes encoding multiple K^+^ channels were downregulated while proliferation and senescence-related pathways were activated [59]. In contrast, *Idh2* was not significantly altered in marginal, intermediate, or basal cells. In addition, while our RNA–seq showed that *Sod2* expression was not significantly changed by cisplatin in OHCs (Table S2), intermediate cells showed a significantly upregulated expression of *Sod2* [59]. Intermediate cells resembled OHCs the most in terms of their response to cisplatin, sharing 394 common DEGs, while marginal (*n* = 149) and basal (*n* = 10) cells showed less overlap [59]. A recent single nuclei RNA–seq (snRNA–seq) study identified 159 DEGs in total across the mouse inner ears following cisplatin treatment [60]. In contrast, our targeted bulk RNA–seq (Table S2) identified 3236 DEGs in OHCs. While scRNA–seq is crucial for investigating heterogenous cell populations, our cell-specific bulk RNA–seq allowed capturing low abundance genes such as *Msra, Gpx7/8*, and *Dele1*. Overall, our study provides the first high-resolution transcriptomic mapping of OHCs after cisplatin treatment (Table S2), and its reversal by honokiol (Table S3).

To investigate the upstream genomic regulators, we carried out a computational unbiased TFBS motif enrichment using DEGs obtained from our DEA (Fig. 10). We observed enrichment of important epigenetic regulators such as *Hdac2* (NES = 4.15) and the chromatin looping protein *Ctcf* (*NES* = 4.09) (Fig. 10A). This is exemplified by the presence of multiple *Ctcf* predicted binding sites across the *Aldh2* promoter and downstream loci (Fig. 10C). On the other hand, honokiol is predicted to suppress the ternary complex factor subfamily involving *Elk1* (NES = 4.07), *Elk3* (NES = 3.80), and *Elk4* (NES = 4.46), which are overrepresented in the cisplatin-only group (Fig. 10B). Overall, these data suggest that honokiol-induced otoprotection may involve the modification of the JNK/MAPK pathway.

Lastly, we investigated the baseline expression profile of OHCs which were exposed to honokiol monotherapy to investigate its effect without cisplatin (Fig. 13). Honokiol monotherapy resulted in the upregulation of unique antioxidant genes, such as *Sod2*, which was not observed in other treatment groups. This proactive upregulation may reinforce the cellular defense of OHCs, before cisplatin treatment takes place and introduces mitochondrial stress. Interestingly, *Idh2*, which was downregulated by cisplatin and upregulated by honokiol + cisplatin, was not significantly altered by honokiol monotherapy (*p* = 0.38). This pattern indicates two distinct classes of honokiol-mediated responses: (1) antioxidant genes (e.g., *Sod2, Prdx5* & *Gstk1*) which are upregulated under normal physiological conditions, and (2) stress-dependent group of antioxidant genes (e.g., *Idh2, Idh1* & *Aldh2*) that require the cisplatin background for upregulation.

While *Sod2* mRNA level was upregulated by honokiol monotherapy, the total SOD2 enzymatic activity remained unchanged in cochlear homogenates across control vs. cisplatin and honokiol + cisplatin vs. cisplatin groups. Honokiol and cisplatin treatment across different genotypes (*Sirt3*^−/−^ and *Sirt3*^+/+^) did not affect the SOD2 activity. These results show that SOD2 enzymatic activity may be further influenced by post-transcriptional factors. Crucially, because SOD2 catalytic activity remained unchanged after cisplatin treatment, the suppression of cochlear H_2_O_2_ by honokiol likely adopted alternative non-SOD2 mechanism, such as the restoration of *Idh2*.

One technical limitation in this study is the different ages of mice between RNA–seq conducted at P25 and physiological tests assessed at Days 42–84. P25 mice are developmentally mature, following the beginning of hearing function at P12–14 [61] and hair cell maturation at P18–21 [61,62]. Nevertheless, mitochondrial homeostasis in juvenile OHCs may not completely mirror that of adult mice at Days 42–84. Manual OHC picking is more efficient in younger rodents, as dissociating OHCs requires milder enzymatic treatment which is less damaging to the cells [62–64]. To ensure sufficient yield and high-quality RNA, we established our protocol using Day 25 mice, not Days 42–84 mice.

On the other hand, CIHL builds up gradually over the three-cycle multiple low-dose treatments and becomes significant mainly after the second cycle [13,33,34], necessitating Days 42–84 mice. Therefore, the DEGs identified in our P25 OHCs represent early transcriptomic dysregulation preceding irreversible auditory dysfunction detected later on. Although we acknowledge this limitation, our sequencing data provide important mechanistic insights for developing proactive otoprotective strategies before hearing loss is permanent at Day 84.

Mice with Cdh23c.753 A exhibit age-related hearing loss beginning 3–6 months of age [31,32]. We minimized this effect by completing auditory tests by Day 84. We acknowledge that the underlying *Cdh23* mutation could have affected the measured hearing threshold to a small extent.

An important concern of a potential otoprotective drug is that the protective agents may interfere with chemotherapy in cancer patients. Having shown that honokiol preserves cisplatin’s anti-tumor activity in vivo [13], the present study used a non-tumor mouse model. This allowed establishing a high-resolution transcriptomic mapping of OHCs without the confounding effects associated with tumor burden.

## Supplementary Material

1

2

3

4

5

6

Figure S2

## Figures and Tables

**Fig. 1.**
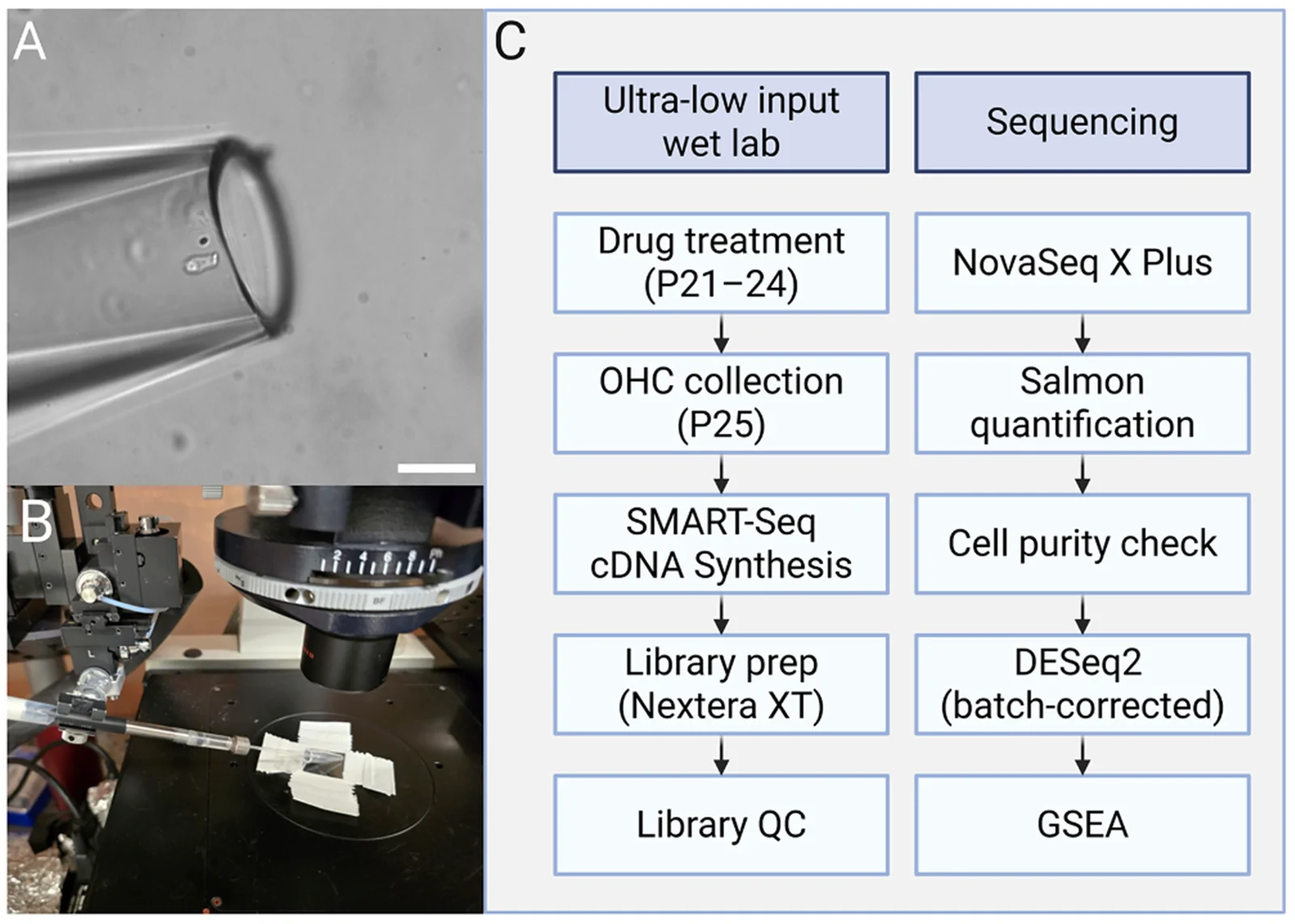
Collecting OHCs for ultra-low input library generation and sequencing**. (A)** An intact OHC was harvested from the cell suspension using fine-tipped glass capillary pipette under a microscope. **(B)** The capillary contents were released into a collection tube while the exit of OHCs from the pipette was visually confirmed. **(C)** Workflow for ultra-low input RNA–seq, including wet lab work and downstream bioinformatic analysis. Scale bar = 50 μm.

**Fig. 2.**
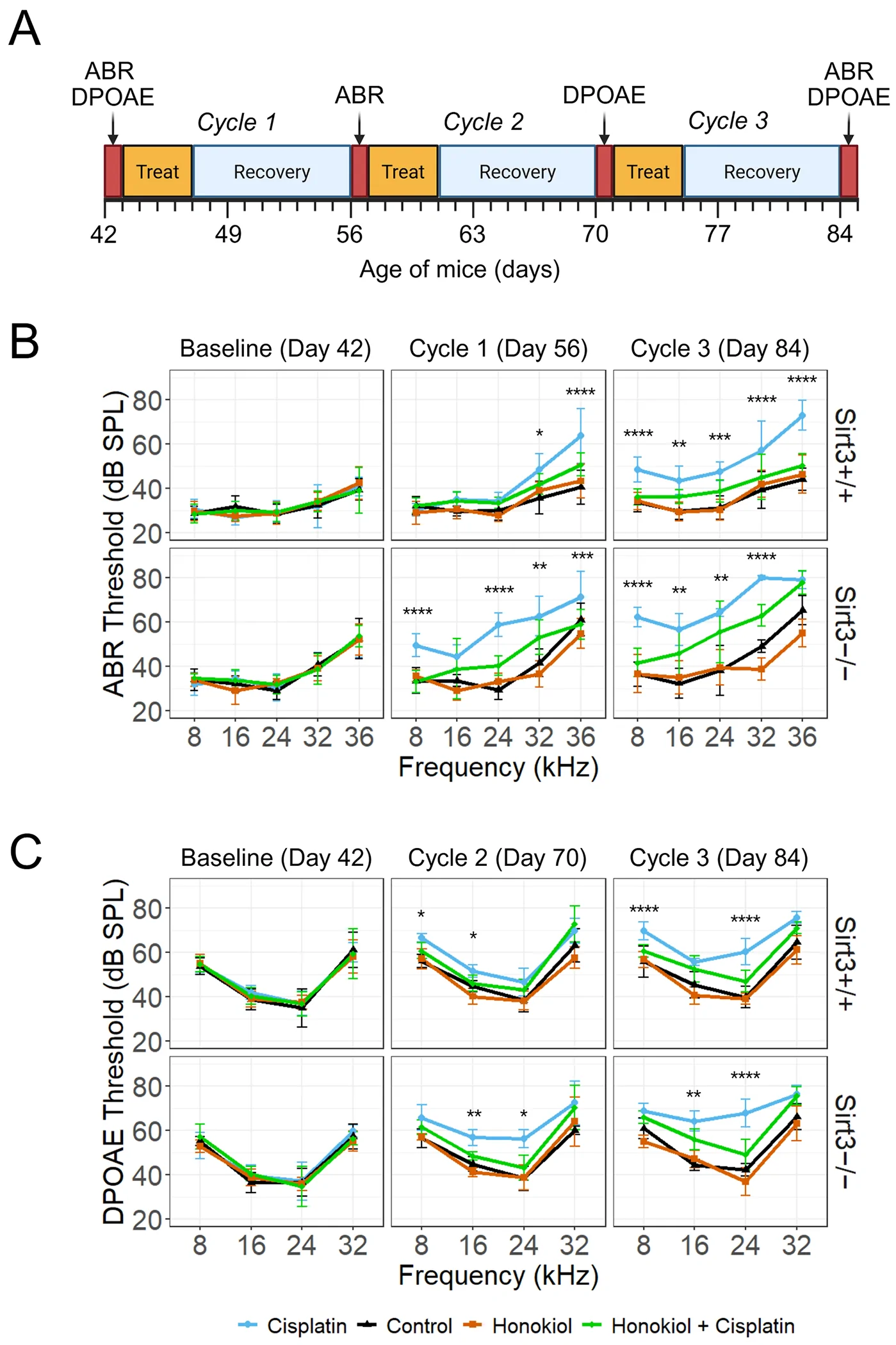
Honokiol attenuates cisplatin-induced ABR and DPOAE threshold elevations in *Sirt3*^+/+^ and *Sirt3*^−/−^ mice.**(A)** Experimental timeline for repeated drug treatments and staggered auditory assessments. Drug treatments began at Day 43. ‘Treat’ indicates four consecutive daily intraperitoneal injections of vehicles, honokiol (10 mg/kg), cisplatin (4 mg/kg), or honokiol pre-treatment + cisplatin. ‘Recovery’ indicates intervals between cycles. Arrows indicate the days on which auditory brainstem response (ABR) and distortion product otoacoustic emission (DPOAE) measurements were performed. **(B)** ABR thresholds were assessed across 8–36 kHz. The time points were Day 42 (baseline), Day 56, and Day 84. Honokiol + cisplatin preserved hearing compared to cisplatin-only in both wild-type (*Sirt3*^+/+^, **Top Row**) and knockout mice (*Sirt3*^−/−^ , **Bottom Row**), especially at Day 56 and Day 84. **(C)** DPOAE thresholds were measured at 8, 16, 24, and 32 kHz. The time points were Day 42 (baseline), Day 70, and Day 84. At Day 42 (baseline), no significant differences were observed between honokiol + cisplatin vs. cisplatin in either genotype. At Day 70, honokiol pre-treatment significantly decreased DPOAE threshold shifts at 8 and 16 kHz for *Sirt3*^+/+^ and 16 and 24 kHz for *Sirt3*^−/−^ . The protection continued at Day 84 in both *Sirt3*^+/+^ and *Sirt3*^−/−^ mice. Data in **(B–C)** are shown as mean ± standard deviation. Linear mixed-effects models were used with fixed effects for genotype, days (week), frequency, and treatment group (honokiol + cisplatin vs. cisplatin alone), with mouse ID as a random effect. Pairwise comparisons (honokiol + cisplatin vs. cisplatin groups) were carried out using estimated marginal means with *p*-values, where the values were adjusted by FDR. *p < 0.05, **p < 0.01, ***p < 0.001, ****p < 0.0001 indicate significant differences between the honokiol + cisplatin vs. cisplatin-only groups. Sample sizes for *Sirt3*^+/+^ mice at baseline **(B–C)**; Control (*n* = 10), Honokiol (*n* = 12), Cisplatin (*n* = 17), Honokiol + Cisplatin (*n* = 17). Sample sizes for *Sirt3*^−/−^ mice at baseline **(B–C)**; Control (*n* = 10), Honokiol (*n* = 10), Cisplatin (*n* = 11), Honokiol + Cisplatin (*n* = 12).

**Fig. 3.**
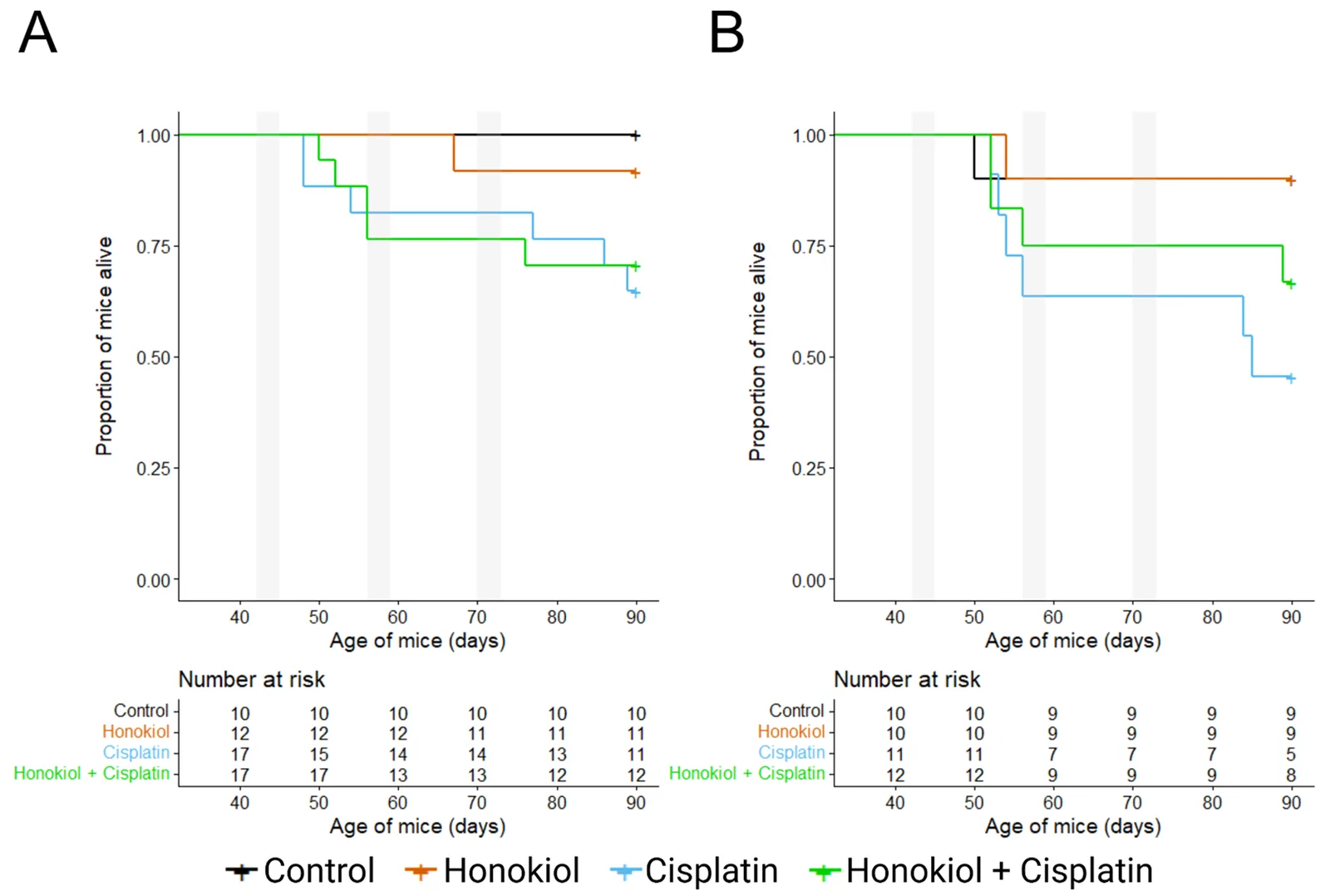
Survival analysis following repeated cisplatin exposure with and without honokiol. Kaplan–Meier survival curves show survival outcomes following repeated cisplatin administrations in **(A)** *Sirt3*^+/+^ and **(B)** *Sirt3*^−/−^ mice. Treatments were administered daily at Days 42–45, 56–59, and 70–73 (gray shaded areas). At Day 90, cisplatin-treated *Sirt3*^−/−^ mice recorded 54.5% survival compared to 75.0% in *Sirt3*^+/+^ mice, without statistically significant difference. Number at risk (mice which were still alive) is shown below each plot.

**Fig. 4.**
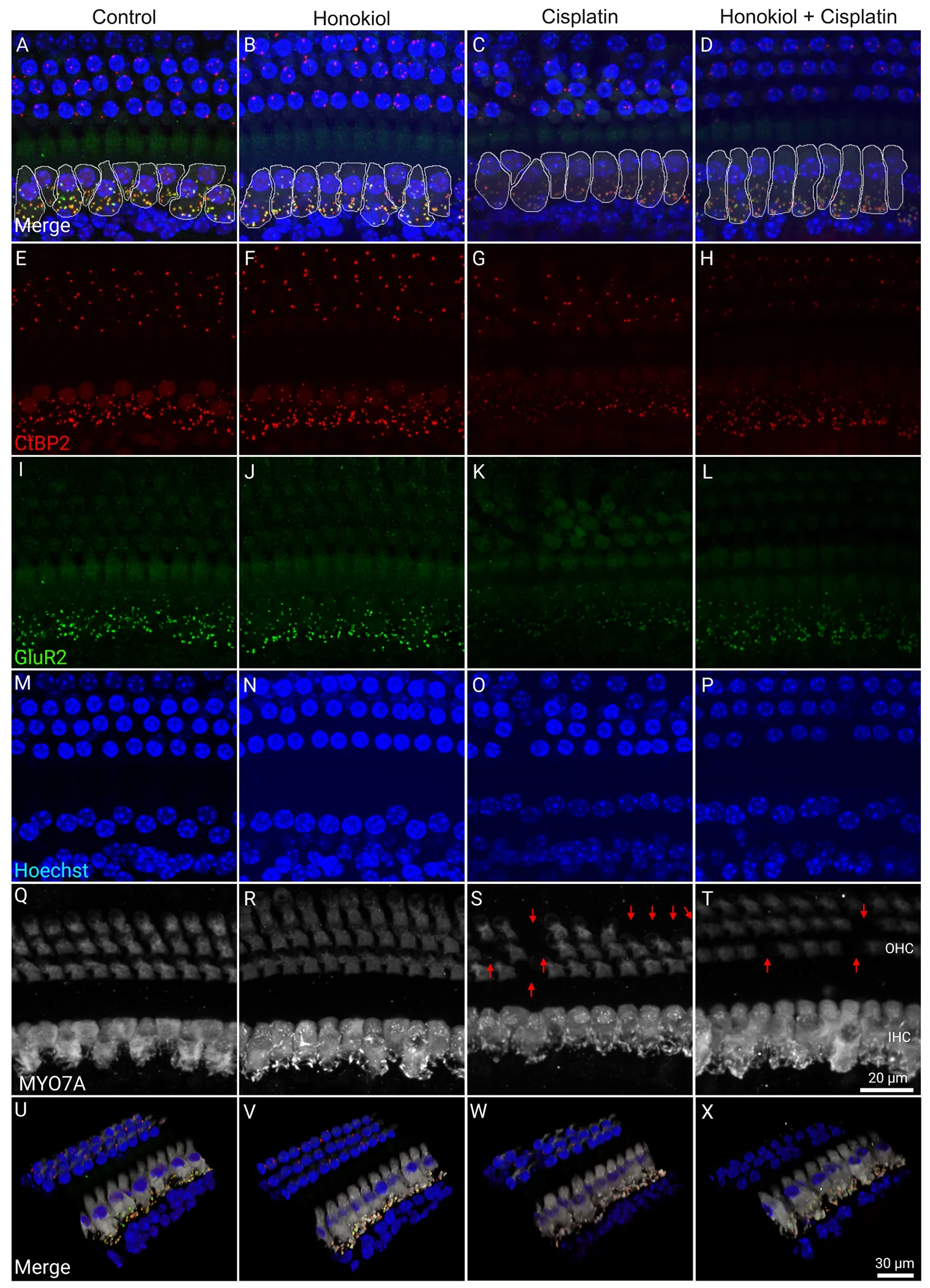
Honokiol protects against cisplatin-induced synaptopathy and outer hair cell loss**.** Confocal maximum intensity projections and 3D renderings of the images taken from the S3 region (17.3–40.0 kHz) of the cochlea from the four experimental groups: control, honokiol, cisplatin, and honokiol + cisplatin. **(A–D)** Merged images showing the spatial organization of hair cells and synaptic markers. **(E–H)** Presynaptic ribbons are labeled with CtBP2 (red). **(I–L)** Postsynaptic densities are labeled with GluR2 (green). **(M–P)** Nuclei are labeled with Hoechst (blue). **(Q–T)** Hair cell bodies are labeled with MYO7A (grayscale). IHC and OHC in **(T)** indicate inner hair cells and outer hair cells, respectively. Red arrows in **(S)** and **(T)** indicate regions of missing OHCs. **(U–X)** 3D surface reconstructions, in which “IHC side” and “OHC side” indicate the anatomical orientation of the organ of Corti. “Synapses” and the accompanying bracket in **(X)** highlight the basolateral cluster of colocalized synaptic puncta. Compared to controls, the cisplatin group visually shows significantly fewer GluR2 puncta and decreased colocalized synapses, while IHCs remain intact in all groups. Honokiol pre-treatment results in an increased survival of OHCs and synaptic integrity. Scale bars = 20 μm **(A–T)**, 30 μm **(U–X)**.

**Fig. 5.**
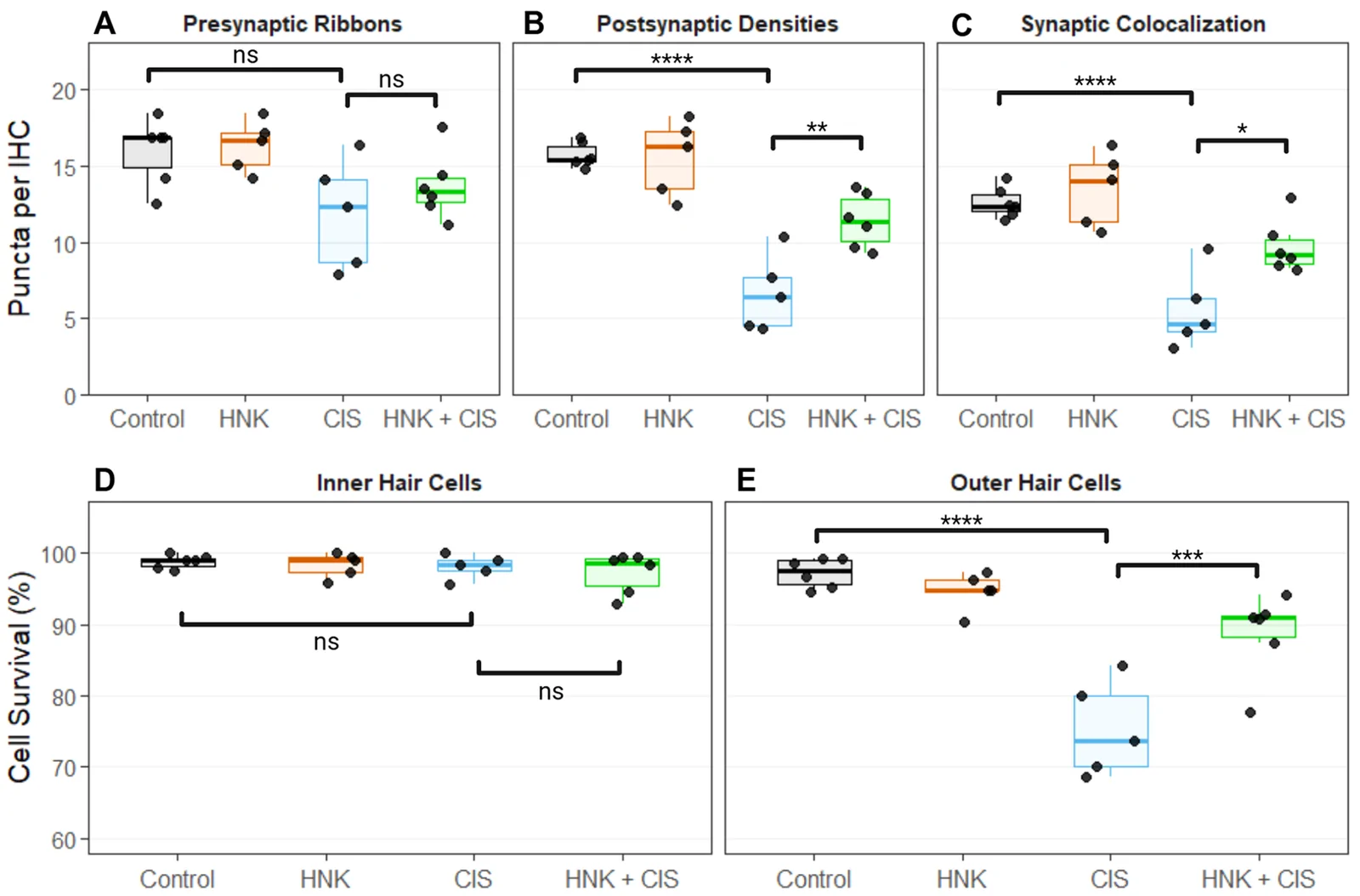
Honokiol ameliorates cisplatin-induced synaptopathy and outer hair cell loss **(A–C)** Synaptic quantification per inner hair cell (IHC). **(A)** Presynaptic ribbons (CtBP2^+^) did not show statistical difference among experimental groups. **(B)** Postsynaptic densities (GluR2^+^) significantly decreased after cisplatin (CIS) treatment, and it was significantly attenuated by honokiol (HNK) pre-treatment. **(C)** Synaptic colocalization (paired CtBP2/GluR2 puncta) was higher in HNK + CIS than CIS. **(D–E)** Hair cell survival analysis. **(D)** IHC counts did not change significantly among treatment groups. **(E)** Outer hair cells (OHCs) showed a decrease in CIS group vs. controls. OHCs showed an increase in the HNK + CIS group vs. CIS group. Statistical Analysis: **A–B** are presented as boxplots showing median, quartiles, and range. Individual data points represent mice. Significance was determined via one-way ANOVA followed by Tukey’s post-hoc test for multiple comparisons. *p < 0.05, **p < 0.01, ***p < 0.001, ****p < 0.0001; ns, not significant.

**Fig. 6.**
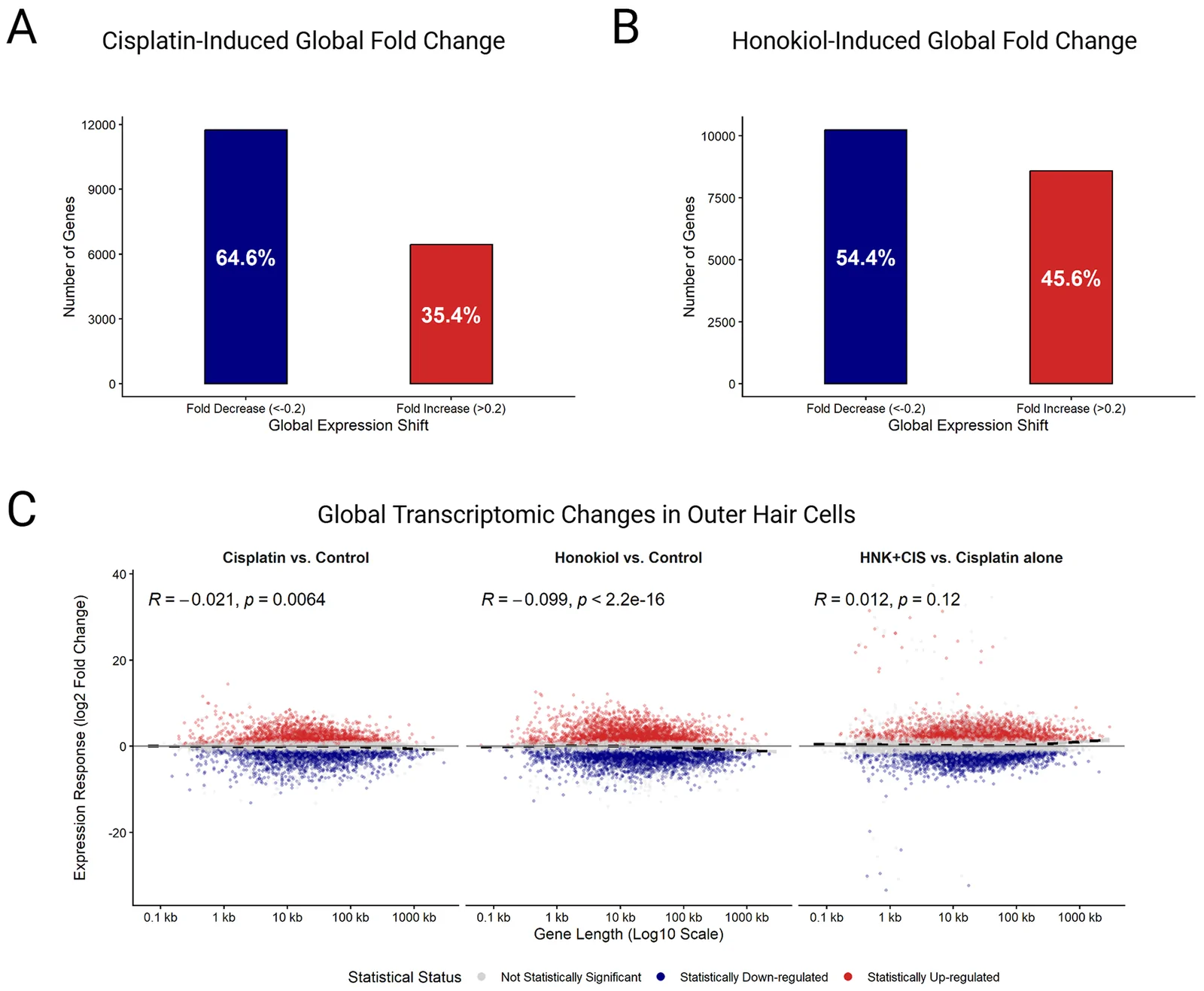
Exploratory global transcriptomic analysis across different gene lengths in outer hair cells. Global transcriptomic distribution and the directional changes in gene expression in mouse outer hair cells in different drug treatment groups. **(A)** Cisplatin-only treatment. Among the 18,196 genes showing a directional change in expression, 64.6% (11,753) showed downregulation (log_2_FC < −0.2, blue) and 35.4% (6443) showed upregulation (log_2_FC > 0.2, red). **(B)** Honokiol-only treatment. Among the 18,822 genes showing a directional change in expression, 54.4% (10,237) showed downregulation (log_2_FC < −0.2, blue) and 45.6% (8585) showed upregulation (log_2_FC > 0.2, red). **(C)** Scatter plot using gene expression changes (log_2_FC) versus genomic gene length (log_10_ scale from 0.1 to 1000 kb) across three comparisons: (1) cisplatin vs. control, (2) honokiol vs. control, and (3) honokiol + cisplatin vs. cisplatin alone. Individual points indicate protein-coding transcripts colored by significant downregulation (blue), significant upregulation (red), and non-significance (grey). Generalized additive model smooth curve (black dashed lines) show the expression trajectory across the gene length scale. Non-parametric Spearman correlation coefficient (*R*) and p-values indicate bias as gene length increases under cisplatin and honokiol treatments, but the trend disappeared in the combination therapy.

**Fig. 7.**
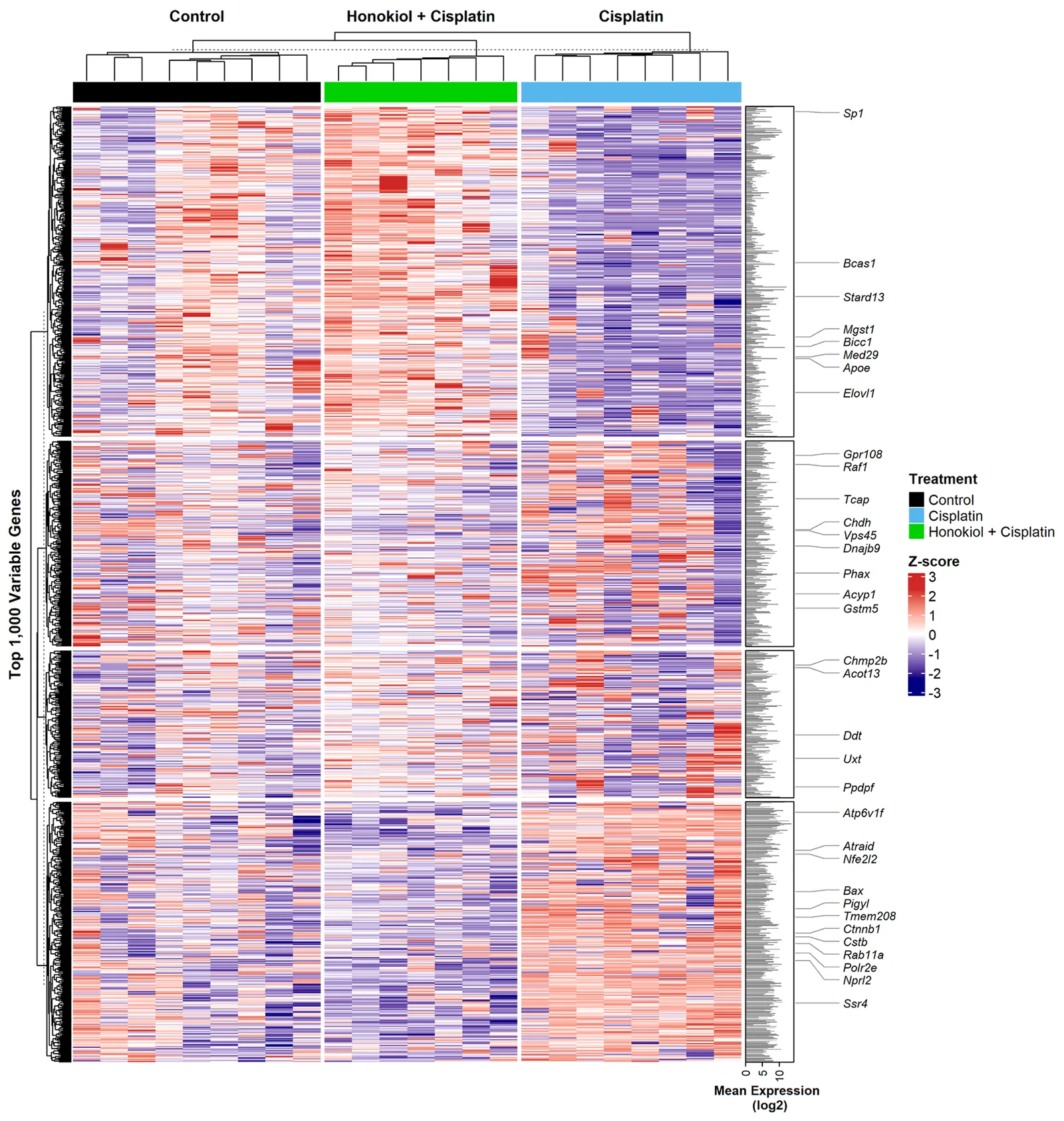
Supervised hierarchical clustering of top variable genes in outer hair cells Supervised hierarchical *k*-means clustering of the top 1000 variable protein-coding genes. Different treatment groups (control, cisplatin, and honokiol pre-treatment + cisplatin) are visualized. Heatmap rows are genes which were selected based on | log_2_FC= > 0.5 between the honokiol + cisplatin and control group, followed by an unsupervised selection finding the remaining highest variance transcripts in the dataset. The genes were filtered to exclude low prevalence reads, predicted genes, pseudogenes, and ribosomal structural proteins. Expressions are shown as a Z-score gradient; blue (low expression), white (neutral), and red (high expression). The horizontal annotation at the top indicates individual biological replicates; control (black), cisplatin (blue), and honokiol + cisplatin (green). Columns are hierarchical clustered. *k* = 4 was used.

**Fig. 8.**
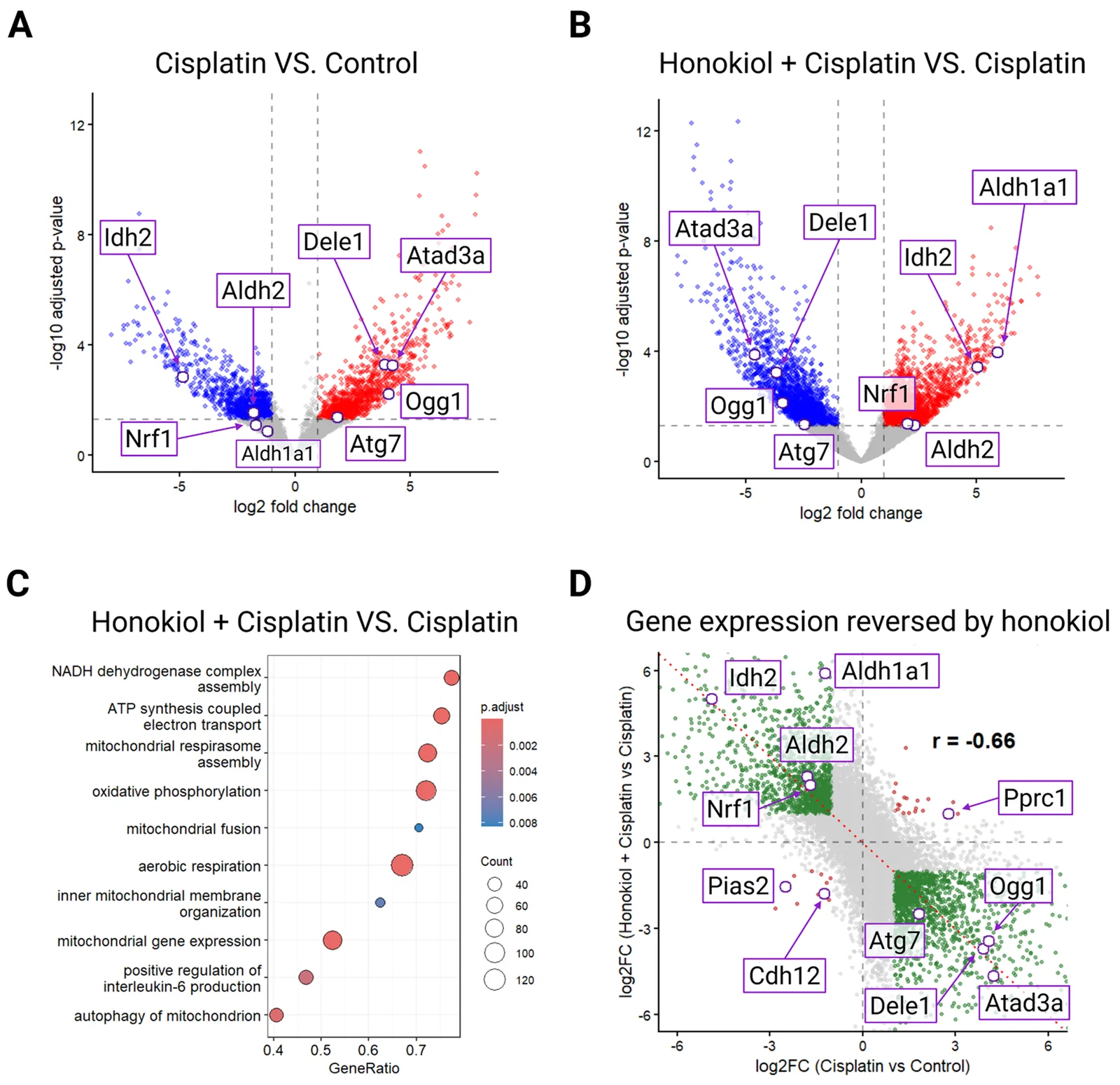
Honokiol reverses cisplatin-induced mitochondrial and stress-response transcriptional changes in outer hair cells**. (A)** Volcano plot showing differential gene expression in outer hair cells after cisplatin treatment. The expressions are relative to controls (vehicles). Cisplatin drove differential expressions of genes involved in mitochondrial function (*Idh2* & *Aldh2*), stress signaling (*Dele1* & *Atad3a*), and autophagy (*Atg7*). Dashed grey lines indicate |log_2_FC= = 1 and *p* = 0.05. **(B)** Differential gene expression comparing honokiol pre-treatment + cisplatin vs. cisplatin alone. Honokiol modulated genes associated with oxidative stress defense (*Aldh1a1*, *Idh2*), mitochondrial regulation (*Dele1* & *Atad3a*), and transcriptional metabolic control (*Nrf1*). **(C)** Gene ontology (GO Biological Process) enrichment analysis of honokiol + cisplatin versus cisplatin alone. Honokiol pre-treatment significantly enriched pathways linked to mitochondrial function. **(D)** Scatter plot showing honokiol-mediated modification of cisplatin-induced damage. It compares log_2_FC induced by cisplatin (x-axis) and those modified by honokiol pretreatment (y-axis). A significant negative correlation (*r* = −0.66) showed that honokiol counteracts cisplatin-induced transcriptional changes. Genes (green) in the 2nd and 4th quadrants represent the reversal of cisplatin effects, while genes (red) in 1st and 3rd quadrants indicate gene expression in which honokiol pre-treatment and cisplatin induce the same directional change. RNA–seq sample sizes: Vehicles (*n* = 9), Honokiol (*n* = 8), Cisplatin (*n* = 8), Honokiol + Cisplatin (*n* = 7). Data are available in GEO database (GSE328003).

**Fig. 9.**
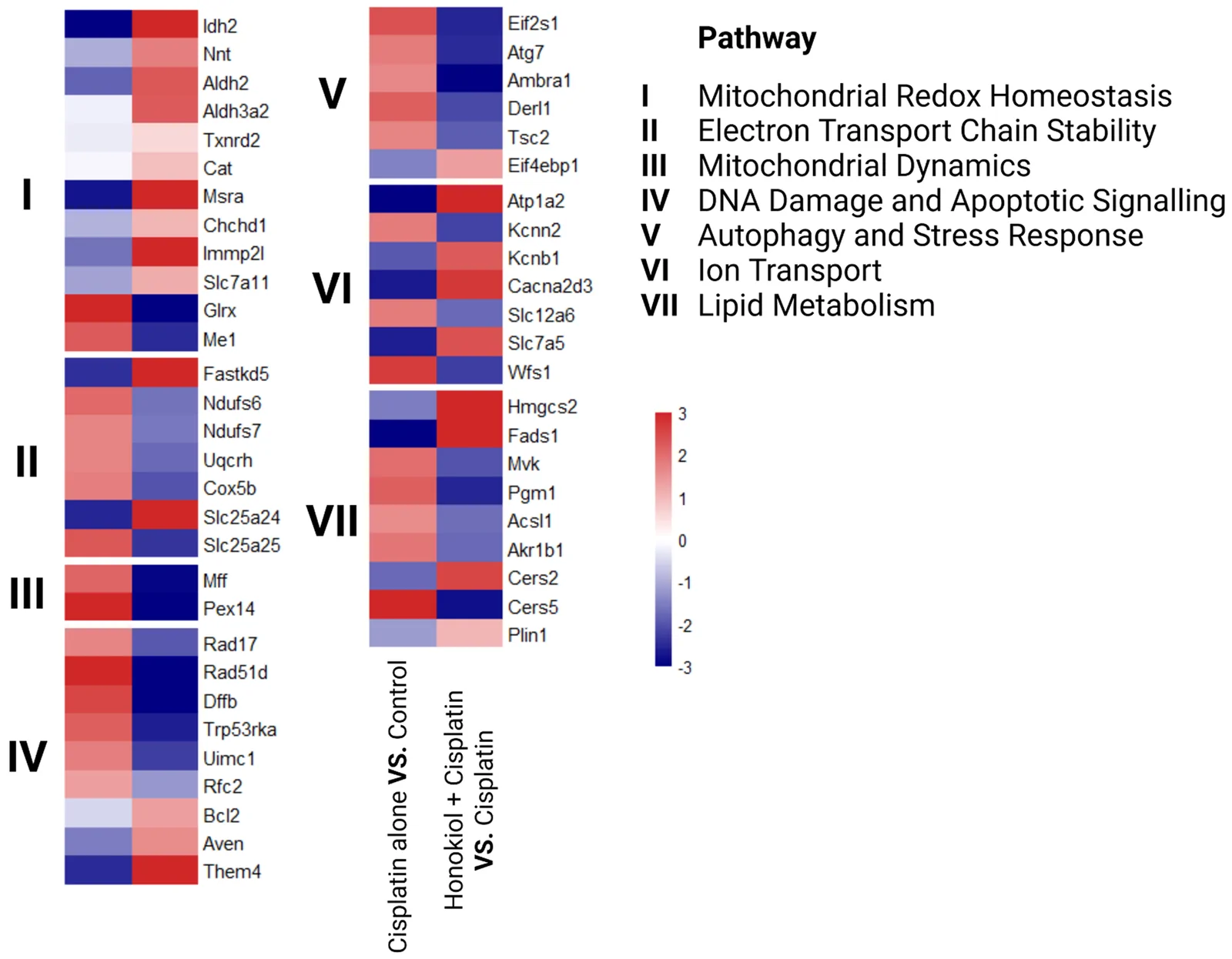
Honokiol reverses cisplatin-induced transcriptional dysregulation across mitochondrial redox, metabolic, and survival pathways in outer hair cells. The heatmaps show genes with bidirectional reversal patterns between: (1) cisplatin vs. controls, (2) honokiol + cisplatin vs. cisplatin. The genes were shown to be significantly altered by cisplatin (*p* < 0.05) and altered by honokiol co-treatment (*p* < 0.05), but in the opposite directions. The genes were limited to protein-coding genes, while predicted genes, RIKEN genes, pseudogenes, and ribosomal genes were excluded. Rows are grouped by biological pathways. Color scale represents log_2_FC (red, upregulated; blue, downregulated). Values are clipped at ±3 for visualization. No hierarchical clustering was applied. Refer to Tables S2–4 **for** lists of differentially expressed genes.

**Fig. 10.**
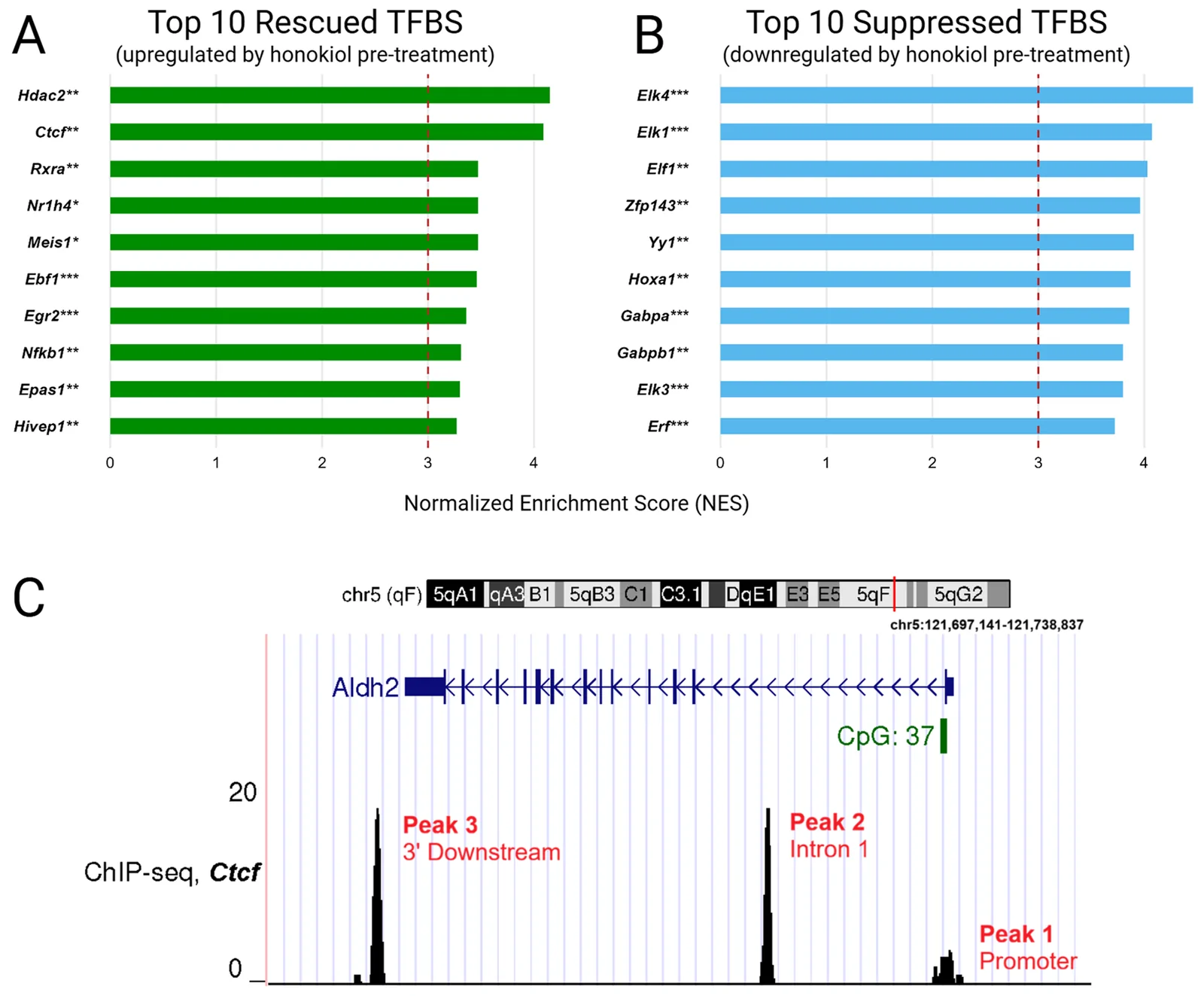
Computational modeling of upstream TFBS enrichment. (A)Motif enrichment analysis showing the top 10 transcription factor binding sites (TFBS). This shows the networks which were significantly downregulated by cisplatin but upregulated by honokiol pre-treatment. **(B)** The top 10 TFBS networks which were upregulated by cisplatin but downregulated by honokiol pre-treatment. Horizontal green and blue bars indicate the maximum normalized enrichment score (NES) measured across the target promoters. The red dashed line indicates NES = 3. *** direct murine annotation. **Inferred cross-mammalian orthology. *Canonical profile. **(C)** An exemplary UCSC genome browser track of *Aldh2* showing the predicted *Ctcf* binding pattern across the promoter, 1st intron, and 3’ downstream region.

**Fig. 11.**
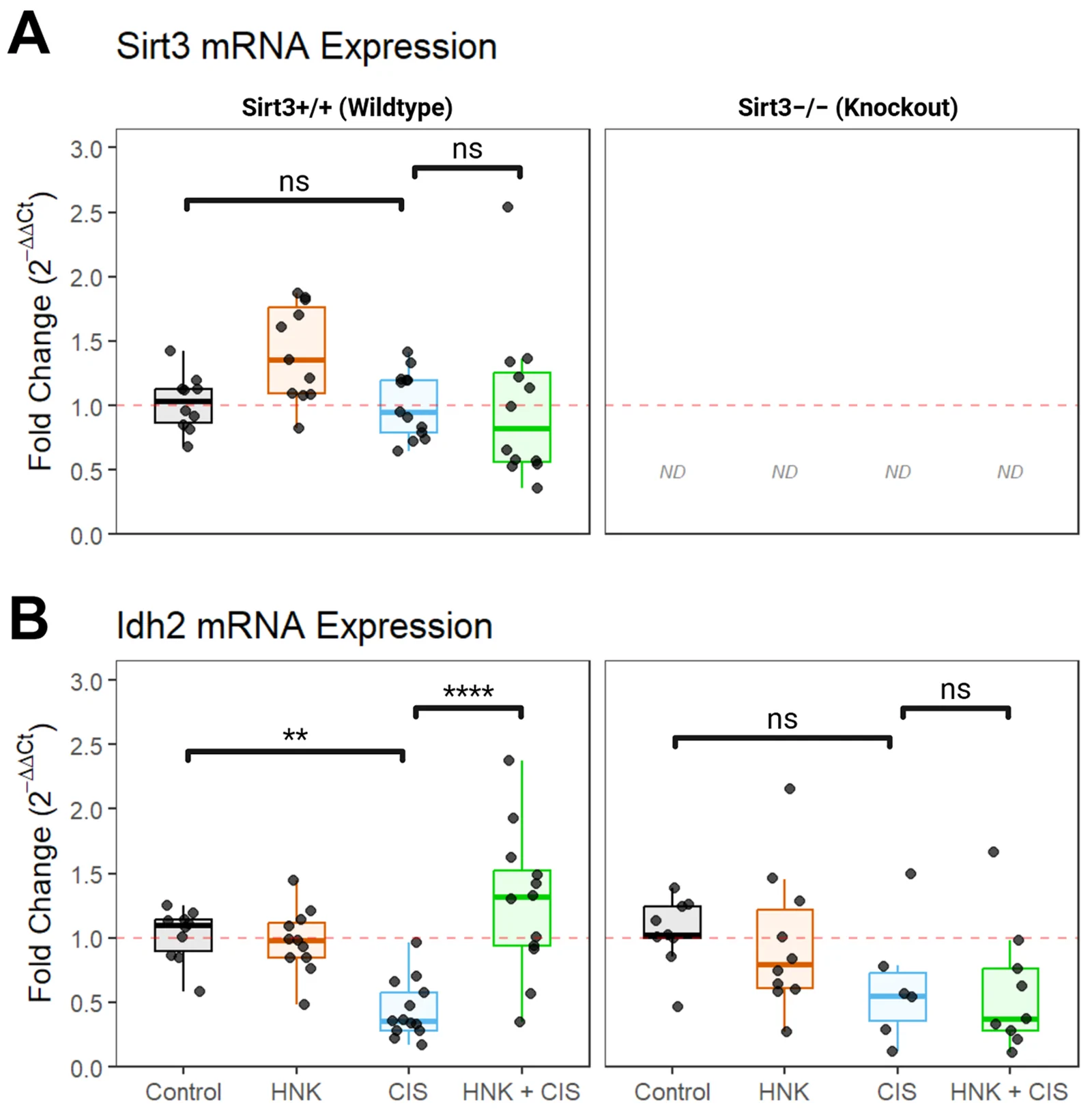
RT–qPCR analysis of honokiol-mediated reversal of cochlear *Idh2* expression through SIRT3-dependent mechanism **(A)** Relative *Sirt3* mRNA expression was measured by RT–qPCR in the cochleae of Day 90 mice. Treatment groups included control, honokiol (HNK), cisplatin (CIS), and HNK pre-treatment + CIS (HNK + CIS). Control vs. CIS did not show a significant change in *Sirt3* expression. HNK + CIS vs. CIS also did not show a significant change. *Sirt3* expression was not detected (*ND*) in *Sirt3*^−/−^ mice. **(B)** Relative *Idh2* mRNA expression was measured across the same treatment groups. In wild-type mice, CIS treatment resulted in a significant *Idh2* downregulation which was reversed in the HNK + CIS group; in *Sirt3*^−/−^ mice, this pattern was not observed. Data are presented as fold change relative to wild-type controls (red dashed line = 1.0). Each data point represents an individual mouse. Boxes indicate the interquartile ranges while horizontal bars represent the means. Statistical significance was determined by Two-way ANOVA (for *Idh2*) or one-way ANOVA (for *Sirt3*) followed by Tukey’s post-hoc test for multiple comparisons. ** *p* < 0.01, **** *p* < 0.0001, ns = not significant.

**Fig. 12.**
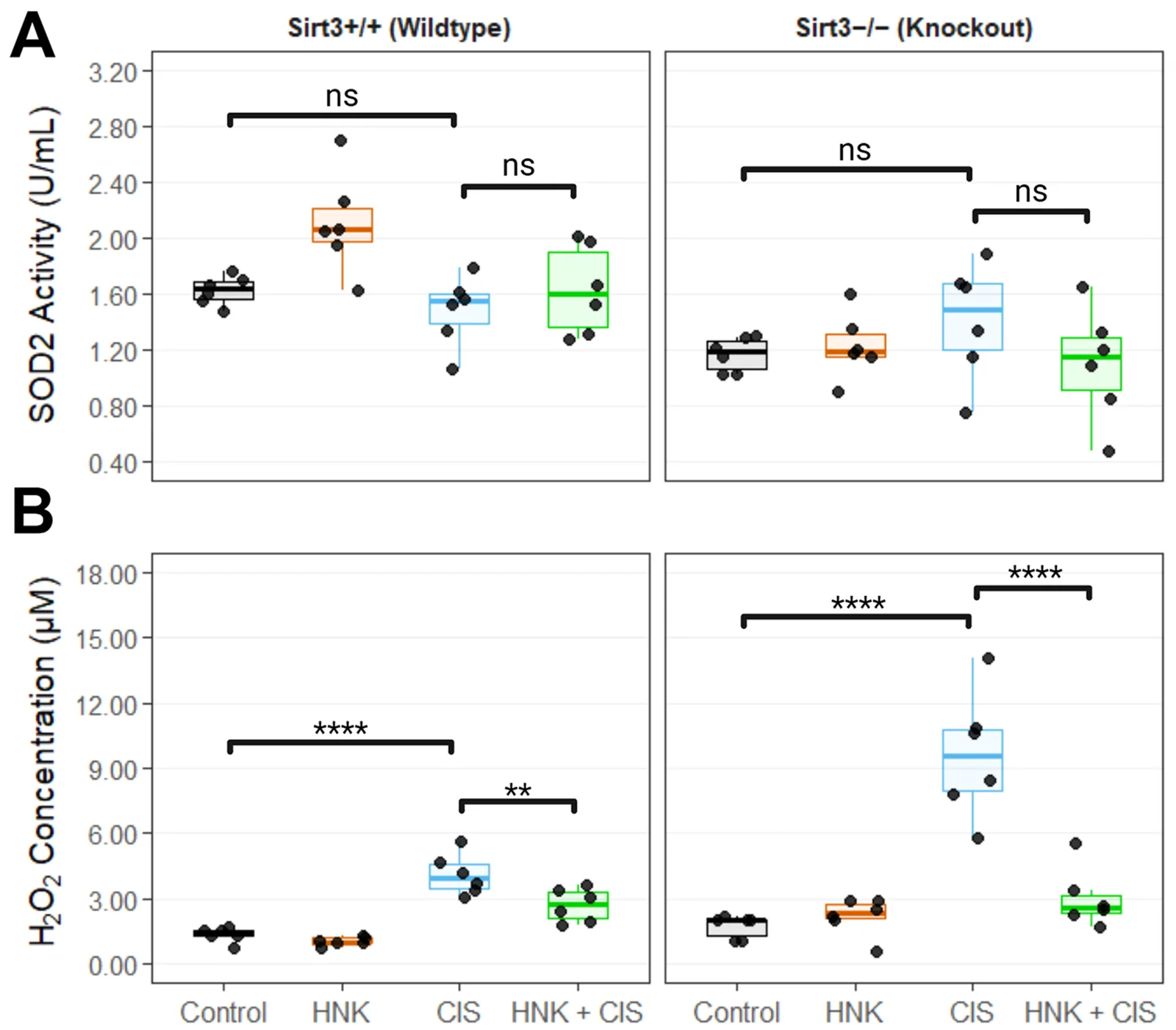
Honokiol attenuates cisplatin-induced oxidative stress in the cochlea**. (A)** Enzymatic activity of mitochondrial superoxide dismutase (SOD2) across treatment groups in wild-type and *Sirt3*^−/−^ (knockout) mice. Treatment groups included control, honokiol (HNK), cisplatin (CIS), and HNK pre-treatment + CIS (HNK + CIS). In both genotypes, no statistically significant differences were observed in CIS vs. control, or in HNK + CIS vs. CIS. **(B)** Concentration of hydrogen peroxide (H_2_O_2_) as a measure of reactive oxygen species (ROS). CIS treatment resulted in a significant increase in H_2_O_2_ levels compared to controls in both genotypes. Co-treatment with HNK (CIS vs. HNK + CIS) significantly decreased H_2_O_2_ concentrations. **A–B** are presented as boxplots showing median, quartiles, and range. Individual data points represent mice. Statistical significance was determined by Two-way ANOVA followed by Tukey’s post-hoc test. Significance levels are shown as ns (not significant), **p < 0.01, and ****p < 0.0001.

**Fig. 13.**
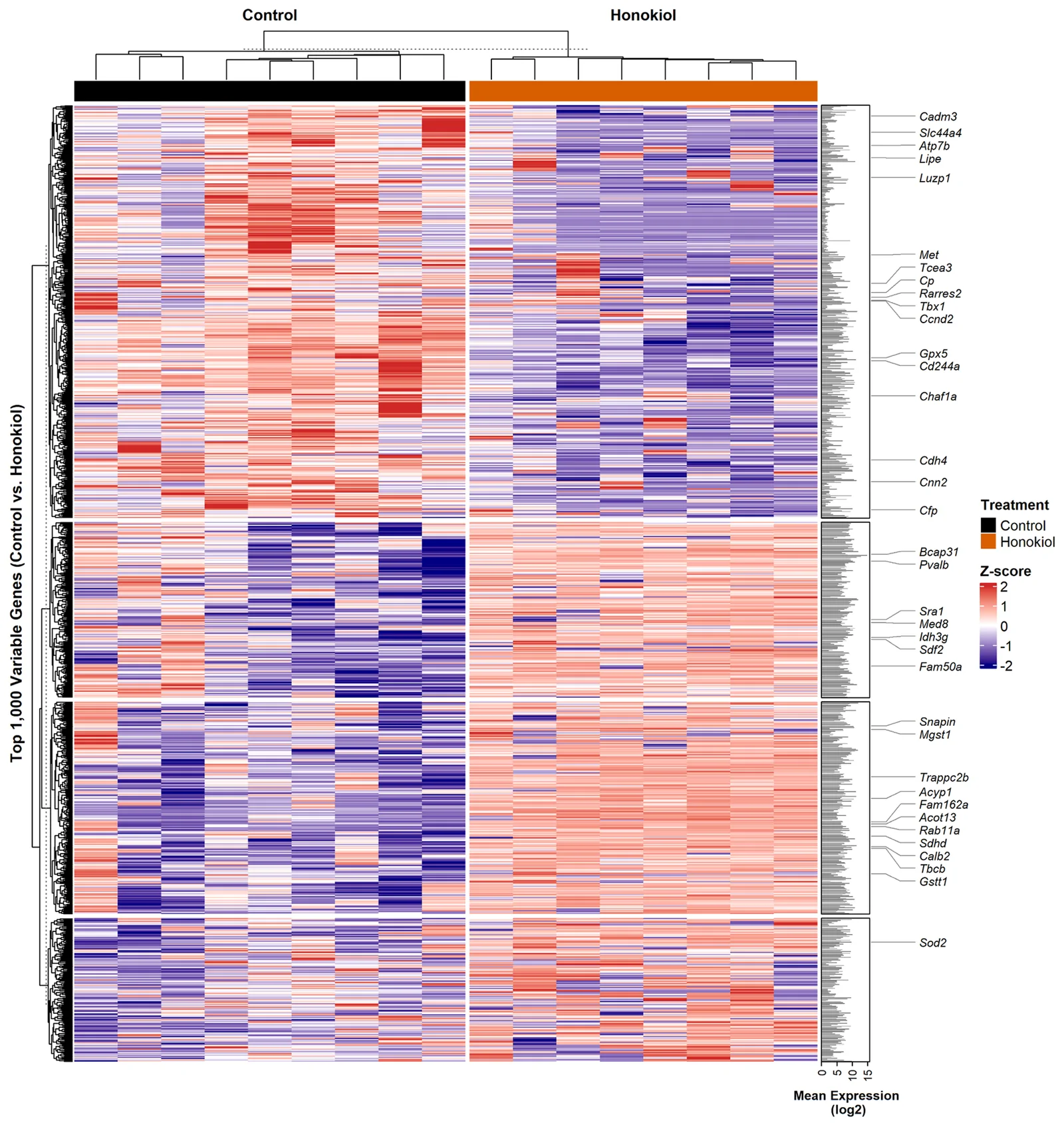
Supervised hierarchical clustering of top variable genes modified by honokiol alone in outer hair cells. Supervised hierarchical *k*-means clustering of the top 1000 variable protein-coding genes. Different treatment groups (control and honokiol) are visualized. Heatmap rows are genes which were selected based on | log_2_FC= > 0.5 between the honokiol and control group, followed by an unsupervised selection finding the remaining highest variance transcripts in the dataset. The genes were filtered to exclude low prevalence reads, predicted genes, pseudogenes, and ribosomal structural proteins. Expressions are shown as a Z-score gradient; blue (low expression), white (neutral), and red (high expression). The horizontal annotation at the top indicates individual biological replicates; control (black) and honokiol (brown). Columns are hierarchical clustered. *k* = 4 was used.

**Table 1:** Antibodies used for synapse immunostaining.

Description	Concentration	Product source	RRID
Mouse IgG1 anti-CtBP2	1:200	Sigma, 612044	AB_399431
Mouse IgG2a anti-GluR2	1:2000	Sigma, MAB397	AB_2113875
Rabbit anti-Myosin VIIA	1:200	Proteus, 256790	AB_2282417
Goat anti-mouse IgG2a AF488	1:3000	Thermo Fisher, A21131	AB_2535771
Goat anti-mouse IgG1 AF568	1:500	Invitrogen, A21124	AB_2535766
Goat anti-rabbit IgGH-L AF647	1:500	Invitrogen, A32733	AB_2633282
Hoechst 33342	1:500	BD Pharmingen, 561908	AB_10626776

Primary and secondary antibodies were used to label presynaptic ribbons (CtBP2), postsynaptic densities (GluR2), and hair cells (MYO7A) in cochlear tissue. The table includes antibody description, working concentration, product source, and research resource identifier (RRID)

## Data Availability

FASTQ files, processed data, and metadata are available in GEO database (GSE328003) with the secure token (adajuyiafnwptwb). Table S1 includes raw data and statistical analysis used in each figure.

## Appendix A. Supporting information

Supplementary data associated with this article can be found in the online version at doi:10.1016/j.biopha.2026.119857.
