# Supplementary material for: Honokiol-mediated reversal of cisplatin-induced transcriptomic changes in outer hair cells
Source: Biomed Pharmacother. Author manuscript; Available in PMC 2026 Oct 15. (PMC13662932; doi:10.1016/j.biopha.2026.119857)
Supplement: Supplementary file 1 [file NIHMS2212988-supplement-1.docx]

**Figure S1** Principal component analysis

Two-dimensional PCA plot visualizing RNA–seq samples (*n* = 32) across different treatments. Transcript counts were imported via tximport and low-expression genes were removed. DESeq2 modeled differential expression using ~ batch + treatment. VST-transformed counts were batched-corrected before PCA on the top 1,000 variable genes.
