# Supplementary material for: Honokiol-mediated reversal of cisplatin-induced transcriptomic changes in outer hair cells
Source: Biomed Pharmacother. Author manuscript; Available in PMC 2026 Oct 15. (PMC13662932; doi:10.1016/j.biopha.2026.119857)
Supplement: Supplementary file 2 [file NIHMS2212988-supplement-2.docx]

**Figure S3** Venn diagram showing significant differentially expressed genes

Pairwise intersection was mapped, comparing significant differentially expressed genes (DEG, p < 0.05) between honokiol vs. control (n = 6,892) and honokiol + cisplatin vs. cisplatin alone (n = 4,933) in mouse outer hair cells. Overlapping segment showed 2,487 transcripts. Left segment (4,446) indicates the baseline response of healthy outer hair cells to honokiol alone. Center segment (2,487) indicates the effect of honokiol regardless of whether the cisplatin background is present or not. Right segment (2,446) indicates the genes which do not change expression under the normal circumstances, but only change under the cisplatin-induced stress.
