# Supplementary material for: Honokiol-mediated reversal of cisplatin-induced transcriptomic changes in outer hair cells
Source: Biomed Pharmacother. Author manuscript; Available in PMC 2026 Oct 15. (PMC13662932; doi:10.1016/j.biopha.2026.119857)
Supplement: Supplementary file 3 [file NIHMS2212988-supplement-Figure_S2.docx]

**Figure S2**. Unadjusted confocal microscope image

Confocal maximum intensity projections and 3D renderings of the images taken from the S3 region (17.3–40.0 kHz) of the cochlea from the four experimental groups: control, honokiol, cisplatin, and honokiol + cisplatin. (A–D) Merged images showing the spatial organization of hair cells and synaptic markers. (E–H) Presynaptic ribbons are labeled with CtBP2 (red). (I–L) Postsynaptic densities are labeled with GluR2 (green). (M–P) Nuclei are labeled with Hoechst (blue). (Q–T) Hair cell bodies are labeled with MYO7A (grayscale). IHC and OHC in (T) indicate inner hair cells and outer hair cells, respectively. Red arrows in (S) and (T) indicate regions of missing OHCs. (U–X) 3D surface reconstructions, in which “IHC side” and “OHC side” indicate the anatomical orientation of the organ of Corti. “Synapses” and the accompanying bracket in (X) highlight the basolateral cluster of colocalized synaptic puncta. Compared to controls, the cisplatin group visually shows significantly fewer GluR2 puncta and decreased colocalized synapses, while IHCs remain intact in all groups. Honokiol pre-treatment results in an increased survival of OHCs and synaptic integrity. Scale bars = 20 µm (A–T), 30 µm (U–X)
